# SopF, a phosphoinositide binding effector, promotes the stability of the nascent *Salmonella*-containing vacuole

**DOI:** 10.1371/journal.ppat.1007959

**Published:** 2019-07-24

**Authors:** Nicole Lau, Amanda L. Haeberle, Brittany J. O’Keeffe, Eleanor A. Latomanski, Jean Celli, Hayley J. Newton, Leigh A. Knodler

**Affiliations:** 1 The Department of Microbiology and Immunology at the Peter Doherty Institute for Infection and Immunity, University of Melbourne, Melbourne, Victoria, Australia; 2 Paul G. Allen School for Global Animal Health, College of Veterinary Medicine, Washington State University, Pullman, WA, United States of America; Stanford University School of Medicine, UNITED STATES

## Abstract

The enteric bacterial pathogen *Salmonella enterica* serovar Typhimurium (*S*. Typhimurium), utilizes two type III secretion systems (T3SSs) to invade host cells, survive and replicate intracellularly. T3SS1 and its dedicated effector proteins are required for bacterial entry into non-phagocytic cells and establishment and trafficking of the nascent *Salmonella*-containing vacuole (SCV). Here we identify the first T3SS1 effector required to maintain the integrity of the nascent SCV as SopF. SopF associates with host cell membranes, either when translocated by bacteria or ectopically expressed. Recombinant SopF binds to multiple phosphoinositides in protein-lipid overlays, suggesting that it targets eukaryotic cell membranes via phospholipid interactions. In yeast, the subcellular localization of SopF is dependent on the activity of Mss4, a phosphatidylinositol 4-phosphate 5-kinase that generates PI(4,5)P_2_ from PI(4)P, indicating that membrane recruitment of SopF requires specific phospholipids. Ectopically expressed SopF partially colocalizes with specific phosphoinositide pools present on the plasma membrane in mammalian cells and with cytoskeletal-associated markers at the leading edge of cells. Translocated SopF concentrates on plasma membrane ruffles and around intracellular bacteria, presumably on the SCV. SopF is not required for bacterial invasion of non-phagocytic cells but is required for maintenance of the internalization vacuole membrane as infection with a *S*. Typhimurium Δ*sopF* mutant led to increased lysis of the SCV compared to wild type bacteria. Our structure-function analysis shows that the carboxy-terminal seven amino acids of SopF are essential for its membrane association in host cells and to promote SCV membrane stability. We also describe that SopF and another T3SS1 effector, SopB, act antagonistically to modulate nascent SCV membrane dynamics. In summary, our study highlights that a delicate balance of type III effector activities regulates the stability of the *Salmonella* internalization vacuole.

## Author summary

Pathogenic bacteria that adopt an intracellular lifestyle must create a specialized niche that supports their replication while avoiding detection and killing by the host. The foodborne pathogen, *S*. Typhimurium, invades epithelial cells lining the intestine and establishes residence within a host-derived membrane-bound compartment called the *Salmonella*-containing vacuole (SCV). To create this intracellular niche, *S*. Typhimurium relies on two type III secretion systems (T3SS). These are specialized needle-like devices that inject bacterial effector proteins into host cells, and thereby manipulate host cell signalling and immune responses. T3SS1 delivers effectors that are required for early events in the infectious cycle. In this study we describe a new T3SS1 effector called SopF that is unique to *Salmonella* spp. We show that SopF associates with host cell membranes by binding to phosphoinositides, which are specialized lipids present in eukaryotic cellular membranes, and that SopF is required for maintaining the integrity of the nascent SCV membrane. *Salmonella* has therefore evolved to reside within a membrane-bound compartment by acquiring a unique type III effector whose actions promote vacuole stability.

## Introduction

Many pathogenic bacteria of public health significance undergo an intracellular cycle as part of their virulence strategy. The ability of these bacteria to direct themselves to a specific intracellular locale is key to their pathogenesis, not only determining their survival and proliferation, but ultimately their virulence. Once internalized, a bacterium can either remain confined within a membrane-bound compartment or lyse its nascent phagosome and colonize the eukaryotic cytosol. The fundamental processes governing intracellular niche selection are poorly understood.

*Salmonella enterica* serovar Typhimurium (*S*. Typhimurium), a common cause of foodborne gastroenteritis, is a facultative intracellular pathogen that can colonize epithelial cells, dendritic cells, macrophages and fibroblasts. Within these cells, *S*. Typhimurium resides within a membrane-bound compartment known as the *Salmonella*-containing vacuole (SCV) [1], that extensively, but selectively, interacts with the host cell endocytic pathway [2]. A critical virulence determinant for *S*. Typhimurium is a specialized injection device, the type III secretion system (T3SS). T3SSs are needle-like multi-protein complexes that penetrate host cell membranes to inject bacterial proteins, termed type III effectors, directly into the host cell cytosol. *S*. Typhimurium translocates ~40 effector proteins [3–6] using two T3SSs, T3SS1 and T3SS2, which are encoded on *Salmonella* Pathogenicity Island (SPI)-1 and SPI-2, respectively. Based upon their timing of expression, T3SS1 effectors are primarily associated with early events in *Salmonella*-host cell interactions such as promoting bacterial entry into non-phagocytic cells and establishment of the nascent SCV [7], whereas T3SS2 effectors contribute to later events including maturation of the SCV and bacterial replication and survival within the host cell, particularly phagocytic cells [8,9].

T3SS1 engages the host plasma membrane to translocate five effectors—SipA, SipC, SopB (also known as SigD), SopE and SopE2—that target the actin cytoskeleton [10] to promote the formation of large lamellipodia-like surface projections, known as membrane ruffles, which engulf and internalize *S*. Typhimurium [11,12]. SopA is also required for the efficient invasion of *S*. Typhimurium, but only in polarized epithelial cell lines and via an unknown mechanism [13]. The T3SS1 effector SptP manipulates signalling networks to counteract the effects of the “entry” effectors and down-regulate plasma membrane ruffling [14]. Another T3SS1 effector, SopD, cooperates with SopB to promote SCV membrane fission from the plasma membrane [15]. Large macropinosomes are formed early after *S*. Typhimurium internalization and these eventually fuse with the nascent SCV [16]—the cooperative actions of SopD and SopB are also required for efficient macropinosome formation [15].

In addition to the plasma membrane, T3SS1 also translocates type III effectors across the nascent SCV membrane and these effectors collectively act to modulate SCV-host endocytic pathway interactions. For example, SopE and SopB mediate recruitment of the GTPase, Rab5, to early SCVs to promote their fusion with early endosomes [17,18]. Rab5 in turn recruits Vps34, a phosphatidylinositol 3-kinase (PI3K), to generate phosphatidylinositol 3-phosphate (PI(3)P) on the nascent SCV [18]. PI(3)P then recruits the SNARE protein VAMP8 [19]. Rab5 and Vps34 recruitment, subsequent PI(3)P production, and VAMP8 recruitment are all dependent on the inositol phosphatase activity of SopB [18,19]. SopB also reduces levels of negatively charged lipids on the nascent SCV, specifically PI(4,5)P_2_ and phosphatidylserine, which alters the surface charge of the vacuole membrane [20] and thereby delays its fusion with late endosomes/lysosomes [21,22]. In support of their important role in post-entry events, numerous T3SS1 effectors (i.e. SopE, SopE2, SipA and SopB) can be detected for many hours post-internalization and/or are required for the efficient replication of bacteria in epithelial cells [22–28].

T3SS needle insertion into bacteria-containing vacuoles is believed to “damage” their membranes. Decoration of damaged vacuoles by host cell galectins acts as a danger signal to target these bacteria for autophagic capture and thus restrict their growth [29–31]. Although typically considered to be a vacuolar pathogen, a sub-population of *S*. Typhimurium lyse their internalization vacuole and escape into the cytosol of mammalian cells in a T3SS1-dependent manner [28,32–34]. *S*. Typhimurium in damaged vacuoles are recognized by the autophagy machinery [30,32]. The frequency of nascent SCV lysis and eventual outcome are dependent upon the cell type. More bacteria lyse their nascent vacuole in epithelial cells (10–20% by 90 min post-infection (p.i.)) compared to macrophages (2–6% by 90 min p.i.) [28,32,34–36], possibly due to bacterial entry being largely driven by T3SS1 in non-phagocytic cells. Once in the cytosol, *S*. Typhimurium hyper-replicate in epithelial cells, whereas in macrophages they do not. This difference might be explained by: (i) incomplete autophagic clearance in epithelial cells, (ii) autophagy-mediated repair of damaged vacuoles in epithelial cells, as has been described for fibroblasts [37], (iii) autophagy supporting cytosolic replication in epithelial cells [38], and/or (iv) higher levels of caspase-1 and caspase-11 inflammasomes in macrophages [36].

If bacterial secretion systems damage vacuole membranes, then why don’t all Gram-negative bacteria that use these injection devices efficiently lyse their vacuoles? We hypothesized that pathogens such as *S*. Typhimurium possess species-specific factors that limit the overall extent of vacuole membrane damage. In support of this hypothesis, here we describe a *Salmonella*-specific type III effector, SopF (SL1344_SL1177), that is translocated by T3SS1 to maintain the integrity of the nascent SCV membrane. Compared to wild type *S*. Typhimurium, a *sopF* deletion mutant showed increased access to the cytosol and association with galectin-8 (GAL8), a marker of vacuole rupture, and p62 and LC3, two autophagy-associated proteins. SopF targets host cell membranes, whether translocated by *S*. Typhimurium or ectopically expressed, and also binds phosphoinositides *in vitro*. We further show that SopF and another T3SS1 effector, SopB, act antagonistically to regulate nascent vacuole membrane dynamics.

## Results

### SopF is translocated by T3SS1

**Fig 1:**
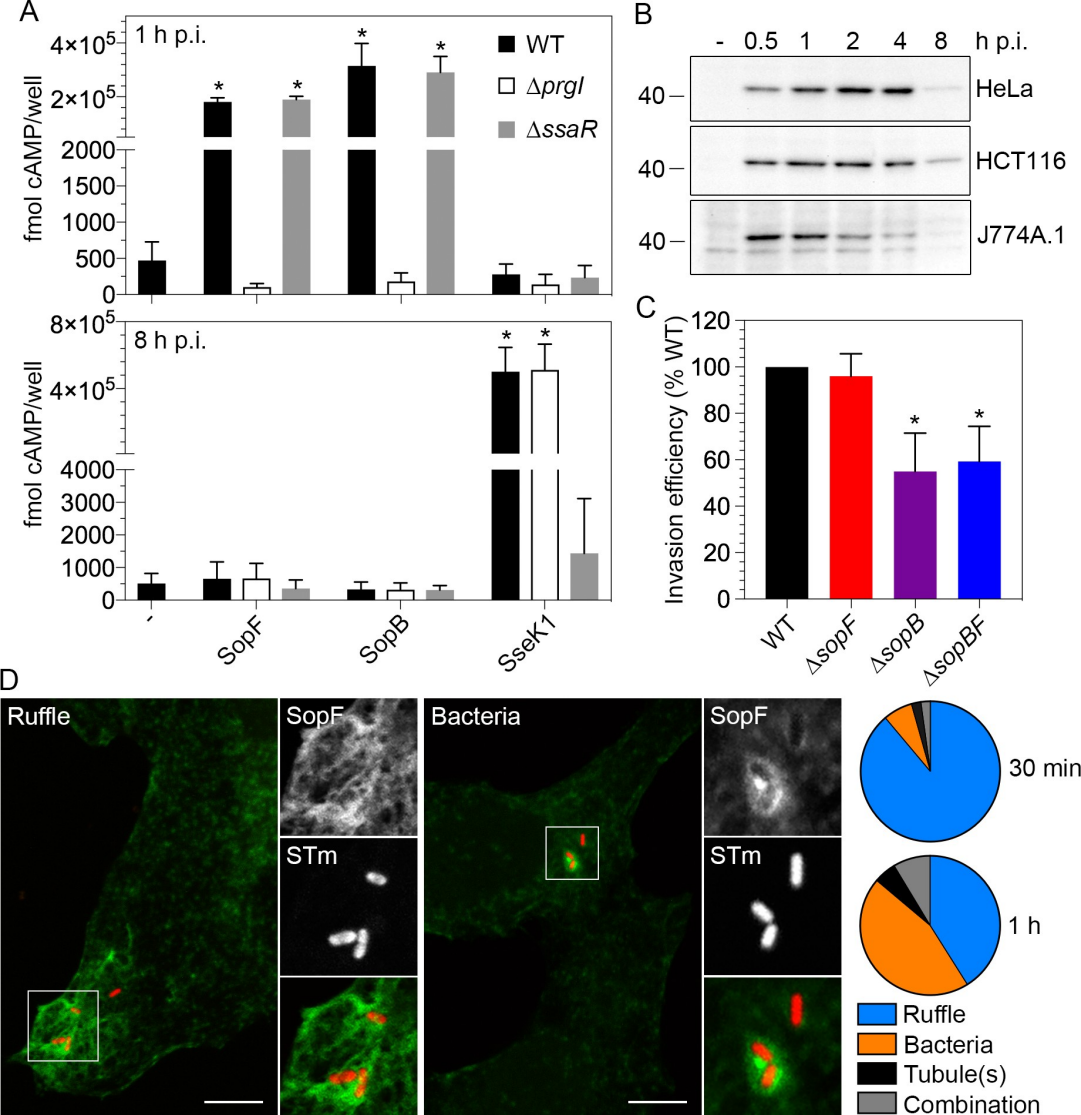
SopF is translocated by T3SS1. **(A)** SopF translocation. J774A.1 macrophage-like cells were infected with *S*. Typhimurium wild type (WT), Δ*prgI*::FRT or Δ*ssaR* strains harboring SopF-CyaA (SopF), SopB-CyaA (SopB) or SseK1-CyaA (SseK1) fusions. Lysates were collected at 1 h and 8 h p.i. and subject to ELISA quantification of cAMP (fmol cAMP/well). Results are the mean ± SD (n≥3 experiments). Asterisks indicate data that is significantly different from WT bacteria with no CyaA plasmid (-) (p<0.05, ANOVA with Dunnett’s post-hoc test). **(B)** Timecourse of intracellular SopF production. HeLa and HCT116 epithelial cells were infected with Δ*sopF* pSopF-3xFLAG bacteria; J774A.1 macrophage-like cells were infected with Δ*sopF* pSopF-2xHA bacteria. Cell lysates were collected at the indicated times p.i., then proteins were separated by SDS-PAGE and subject to immunoblotting with anti-FLAG or anti-HA antibodies. Loading was normalized to equivalent CFU for each timepoint. Molecular mass markers are indicated on the left. (-) indicates uninfected lysate. Results are representative of 2–3 independent experiments. **(C)** SopF is not required for bacterial entry into non-phagocytic cells. HeLa epithelial cells were infected with *S*. Typhimurium wild type (WT), Δ*sopF*, Δ*sopB* or Δ*sopBΔsopF* (Δ*sopBF*) bacteria and invasion efficiency (the percent of inoculum internalized at 1 h p.i.) was quantified by gentamicin resistance assay. The invasion efficiency of *S*. Typhimurium WT was set to 100% for each experiment. Data are the mean ± SD (n = 3 experiments). Asterisks represent data significantly different from WT bacteria (one-way ANOVA with Dunnett’s post-hoc test). **(D)** Translocated SopF localizes to *Salmonella*-induced plasma membrane ruffles and around bacteria. HeLa cells seeded on coverslips were infected with Δ*sopF* pSopF-3xFLAG bacteria (constitutively expressing mCherry) and fixed/permeabilized at 30 min or 1 h p.i. Translocated SopF-3xFLAG was detected using tyramide signal amplification. Representative confocal images of SopF accumulation (shown in green) in plasma membrane ruffles (left panel) or around bacteria, presumably on the SCV (right panel). mCherry bacteria (STm) are shown in red. Scale bars are 10 μm. Insets show enlargements of boxed areas. Pie charts show the mean percentage distribution of SopF localization patterns at 30 min and 1 h p.i. (n≥3 independent experiments). Categories are plasma membrane ruffles, bacteria, membrane tubule(s) originating from the SCV or a combination of these (bacteria plus ruffles, ruffles plus tubule(s) or bacteria plus tubules).

In a RNAseq-based screen for *S*. Typhimurium SL1344 genes that are up-regulated in the cytosol compared to the vacuole during colonization of epithelial cells (T.R. Powers and L.A. Knodler, manuscript in preparation) we identified *SL1344_1177* as a gene that is up-regulated in a subset of cytosolic *Salmonella* at 8 h post-infection (p.i.), a phenotype similar to that described for T3SS1-associated genes [27,33]. *SL1344_1177* is regulated by HilA, HilC and HilD [39] and recent CHIP-seq analysis identified that its counterpart in *S*. Typhimurium 14028s, *STM14_1486*, is a direct target of InvF binding [40]. *SL1344_1177* is therefore part of the SPI-1 regulatory network. SL1344_1177 was recently renamed SopF by Zhou and colleagues [41] (we will adopt this nomenclature henceforth) and is annotated as a “predicted bacteriophage protein”. It is encoded in SPI-11, which is inserted next to the Gifsy-1 prophage and includes a number of genes involved in *Salmonella* pathogenesis [42]. SPI-11 is one of eight core pathogenicity islands present in *Salmonella enterica* subspecies *enterica* (lineage I), which is the subspecies most commonly associated with disease [43,44]. Taken together, this information hinted that SopF could be a candidate T3SS1 translocated effector. To test this, we constructed a fusion of the N-terminal 199 amino acid residues of SopF to the catalytic domain of adenylate cyclase (CyaA) under the control of its native promoter, and electroporated this plasmid into *S*. Typhimurium wild type and two genetic mutants that are defective for T3SS1 translocation (Δ*prgI*) or T3SS2 translocation (Δ*ssaR*). We also utilized bacterial growth conditions that either induce or repress each T3SS [45] and J774A.1 mouse macrophage-like cells as an infection model as they are permissive for the entry of T3SS1 mutants [46,47]. As a readout of SopF-CyaA translocation into host cells, cAMP production was quantified by ELISA. Using late log-phase subcultures, which are induced for T3SS1, we detected robust SopF-CyaA translocation into J774A.1 cells by 1 h p.i.; cAMP production was dependent on a functional T3SS1 but not T3SS2 (Fig 1A, upper panel). To test for T3SS2 dependence, bacteria were grown under conditions where T3SS1 was repressed (stationary phase cultures) and infected cell lysates were collected at 8 h p.i.; no cAMP was produced after infection with bacteria harboring SopF-CyaA or SopB-CyaA fusions (Fig 1A, lower panel). As a positive control, we used the T3SS2 effector, SseK1 [48]. Infection of J774A.1 cells with bacteria harboring an SseK1-CyaA fusion raised intracellular cAMP levels substantially in a manner dependent on *ssaR* (Fig 1A, lower panel). Overall, these data indicate that SopF is a T3SS1 effector, which agrees with a recent study that showed SopF is delivered into host cells [41].

To examine the kinetics of intracellular *sopF* expression, we infected mammalian cells with Δ*sopF* pSopF-3xFLAG or Δ*sopF* pSopF-2xHA bacteria and samples were collected for immunoblotting at various times p.i. Loading of protein samples was normalized to equivalent colony forming units (CFU) over the time course. In epithelial cells (HeLa and HCT116), SopF-3xFLAG was highly produced from 0.5–4 h p.i. and declined thereafter but was still detectable at 8 h p.i (Fig 1B). In J774A.1 macrophage-like cells, the peak of SopF-2xHA production was 0.5–1 h p.i., after which it markedly declined (Fig 1B). These patterns of expression are strikingly similar to that shown for another T3SS1 effector, SopB, in epithelial cells [23] and macrophages [49]. However, unlike what has been shown for SopB [50], we did not detect a role for SopF in the invasion of non-phagocytic cells (Fig 1C). Furthermore, the invasion efficiency of a Δ*sopB*Δ*sopF* mutant was comparable to a Δ*sopB* mutant (Fig 1C), indicating that SopB and SopF do not act cooperatively to promote bacterial internalization. The Δ*sopF* mutant also did not have a replication defect in epithelial cells or macrophages compared to wild type bacteria (S1 Fig). Altogether, these data show that SopF is a T3SS1 effector that is produced early during host cell infection but is not overtly required for the entry of *S*. Typhimurium into host cells or intracellular replication.

To examine the subcellular localization of bacterially translocated SopF, we infected HeLa cells with Δ*sopF* pSopF-3xFLAG bacteria (constitutively expressing mCherry), then fixed, permeabilized and immunostained with anti-FLAG antibodies. Under our fixation-permeabilization conditions, only extrabacterial SopF-FLAG will be accessible to antibodies. No specific FLAG signal was detected in infected cells up to 2 h p.i., suggesting that either the FLAG antigen is masked or SopF is translocated in low quantities. In support of the latter, when we amplified the antibody signal by the addition of tyramide, translocated SopF-3xFLAG could be visualized in ~20% of infected cells at 30 min and 1 h p.i. Thirty minutes after infection, SopF primarily localized to plasma membrane ruffles formed on the dorsal surface of cells in close proximity to internalized bacteria (Fig 1D). By 1 h p.i., SopF also concentrated around intracellular bacteria, presumably decorating the SCV (Fig 1D). On rare occasions, membrane tubules emanating from the SCV were also positive for SopF-3xFLAG signal (Fig 1D). From these observations we conclude that translocated SopF targets a distinct subset of host cell membranes, namely those that are co-opted by *Salmonella* during bacterial internalization and intracellular trafficking.

### Ectopically expressed SopF localizes to eukaryotic cell membranes

**Fig 2:**
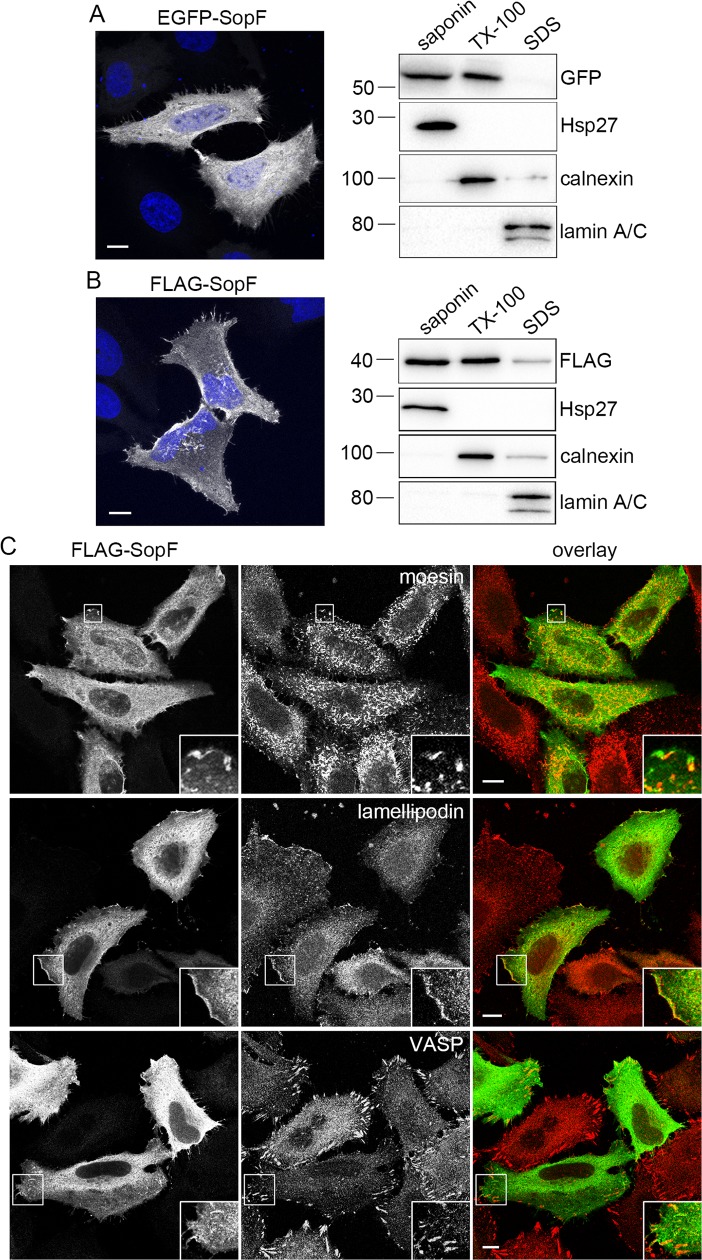
SopF associates with mammalian cell membranes. **(A)** HeLa cells were transfected with a plasmid encoding for EGFP-SopF for 18 h and then subject to confocal fluorescence microscopy or sequential detergent fractionation. Left panel shows a representative confocal microscopy image. EGFP-SopF (greyscale), DNA (blue). Scale bar is 10 μm. Right panel shows immunoblotting analysis. Cells were collected and subject to sequential detergent fractionation. Equal volumes of saponin-soluble, TX-100-soluble, and SDS-soluble fractions were separated by SDS-PAGE and subject to immunoblotting with antibodies against GFP, Hsp27 (cytosol), calnexin (membranes) and lamin A/C (nucleus). Molecular mass markers are indicated on the left. Results are representative of two independent experiments. **(B)** As for (A) except HeLa cells were transfected with a plasmid encoding for FLAG-SopF. FLAG-SopF was detected by immunostaining (left panel) or immunoblotting (right panel) with anti-FLAG antibodies. **(C)** SopF partially colocalizes with actin-binding proteins found at cell adhesion sites. HeLa cells were transfected with pFLAG-SopF for 18 h, then fixed and immunostained with anti-FLAG, anti-moesin, anti-lamellipodin and anti-vasodilator-stimulated phosphoprotein (VASP) antibodies. Representative confocal microscopy images show FLAG-SopF in green and moesin, lamellipodin or VASP in red. Scale bars are 10 μm. Insets show enlargements of boxed areas.

We next used ectopic expression of SopF to gain insight into its biological function. HeLa cells were transfected with plasmids encoding for EGFP-SopF or FLAG-SopF and steady-state protein localization was monitored by fluorescence microscopy. SopF was distributed throughout the cytosol, and also accumulated at the leading edge of cells and in filopodia-like structures extending out from the plasma membrane (Fig 2A and 2B). At the leading edge, FLAG-SopF and EGFP-SopF partially co-localized with actin-binding proteins such as moesin, which is involved in cell adhesion, membrane ruffling and microvilli formation [51], vasodilator-stimulated phosphoprotein (VASP), which regulates filopodial length and dynamics [52] and lamellipodin, a regulator of lamellipodia protrusion and cell migration [53] (Fig 2C, S2A Fig).

We also used biochemical fractionation to determine the subcellular localization of SopF. Transfected HeLa cells were first treated with saponin, which permeabilizes the plasma and internal membranes and releases their soluble content, followed by Triton X-100 (TX-100), which solubilizes integral and peripheral membrane proteins, and lastly SDS to solubilize all remaining cellular components. Samples were subject to immunoblotting with antibodies against GFP, FLAG, Hsp27 (cytosolic protein), calnexin (integral membrane protein) and lamin A/C (nuclear protein). EGFP-SopF (Fig 2A) and FLAG-SopF (Fig 2B) equally partitioned to the saponin- and TX-100-soluble fractions, indicating that SopF is both cytosolic and membrane-associated when ectopically expressed.

### SopF binds phosphoinositides

**Fig 3:**
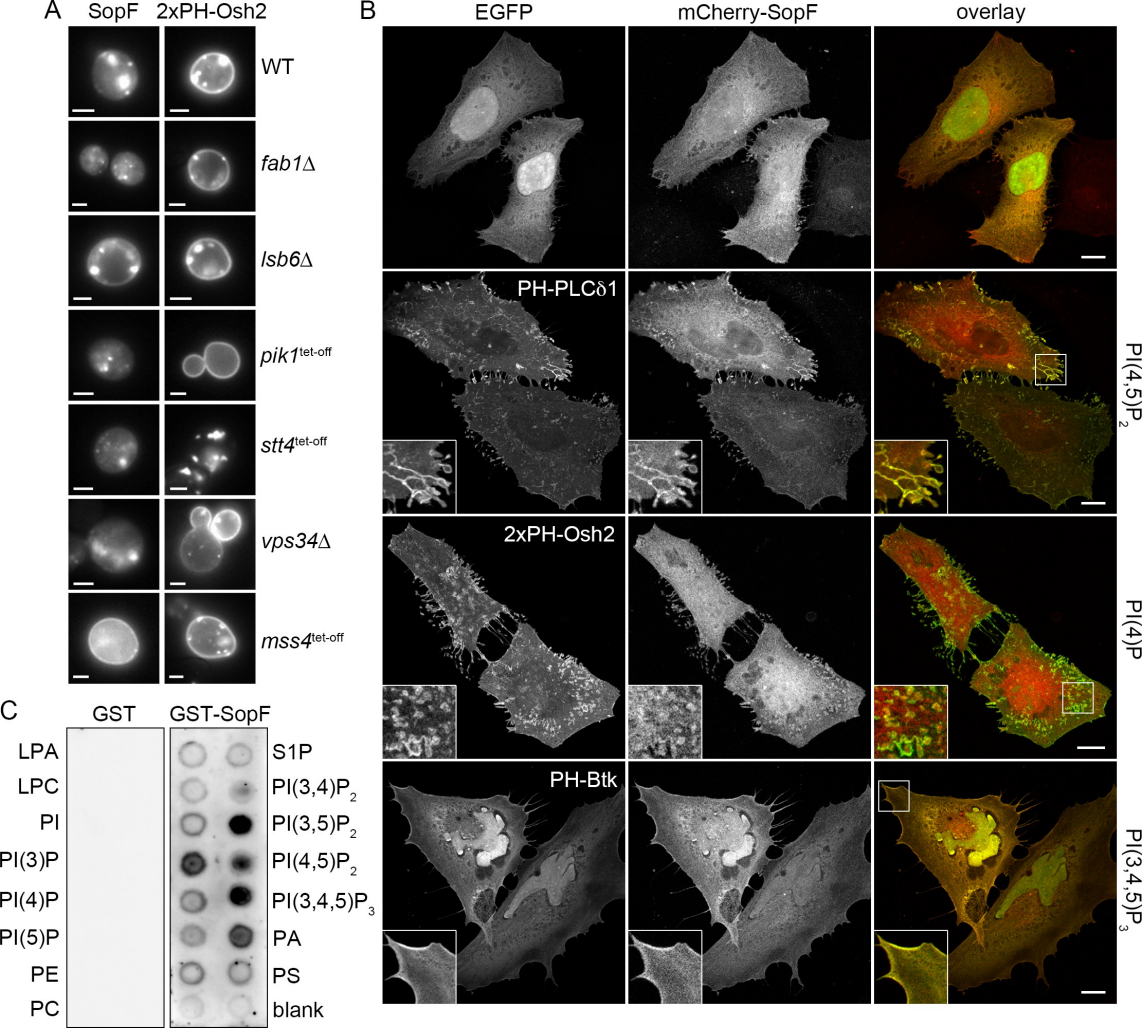
SopF binds phosphoinositides. **(A)** *Saccharomyces cerevisiae* wild type and six mutant PI kinase strains were transformed with yEGFP-SopF. Tet-off strains were repressed for 24 h with doxycycline before the expression of SopF was induced with galactose. The subcellular localization of SopF was monitored in live cells by widefield fluorescence microscopy. SopF relocalizes from internal membrane sites to the plasma membrane in the *mss4*^tet-off^ strain, which has decreased PI(4,5)P_2_ and increased PI(4)P levels at the plasma membrane. yEGFP-2xPH-Osh2, which localizes to the Golgi and plasma membrane in yeast via PI(4)P binding, was used as a control for each strain. Representative fluorescence images are shown. Scale bars are 2 μm. **(B)** SopF colocalizes with multiple phosphoinositide pools present on the plasma membrane in mammalian cells. HeLa cells were co-transfected with mCherry-SopF and EGFP or EGFP-PH domain chimeras for 15 h and fixed. Representative confocal microscopy images show phosphoinositide-binding probes in green and mCherry-SopF in red. PI(4,5)P_2_, PI(4)P, and PI(3,4,5)P_3_ pools at the plasma membrane are bound by PH-PLCδ1, 2xPH-Osh2 and PH-Btk, respectively. Scale bars are 10 μm. Insets show enlargements of boxed areas. **(C)** SopF binds to multiple phosphoinositides *in vitro*. Recombinant glutathione S-transferase (GST) and GST-SopF were purified by affinity chromatography and incubated with PIP Strips (Echelon Biosciences) at 1 μg/ml. Bound GST and GST-SopF were detected using anti-GST antibodies followed by chemiluminescence detection. Compounds spotted on the membrane (100 pmol lipid per spot) are: lysophosphatidic acid (LPA); lysophosphatidylcholine (LPC); phosphatidylinositol phosphate (PI); PI(3)P; PI(4)P; PI(5)P; phosphatidyl ethanolamine (PE); phosphatidyl choline (PC); sphingosine-1-phosphate (S1P); PI(3,4)P_2_; PI(3,5)P_2_; PI(4,5)P_2_; PI(3,4,5)P_3_; phosphatidic acid (PA); phosphatidylserine (PS).

Bacterial effectors often use specialized membrane-localization domains to target to host cell membranes. These include coiled-coil domains, lipidation, ubiquitylation, and phosphoinositide binding [22,25,54–61]. Phosphoinositides are concentrated on the cytosolic surfaces of membranes and the subcellular localization of each phosphoinositide is tightly governed by the combined actions of lipid kinases and lipid phosphatases, giving each cellular membrane a unique and dynamic phosphoinositide signature [62]. To test for a role of phosphoinositides in directing SopF localization, we used a loss-of-function PI kinase screen in the model eukaryotic organism, *Saccharomyces cerevisiae* [58]. Yeast have six main PI kinases that phosphorylate the 3’, 4’ or 5’ position of phosphoinositides and inactivation of a particular PI kinase, via genetic deletion or conditional repression, affects the generation of a specific phosphoinositide pool (S3A Fig). The tandem pleckstrin homology (PH) binding domain of oxysterol-binding protein homolog 2 (Osh2) from *S*. *cerevisiae* was used as a positive control. 2xPH-Osh2 localizes to the Golgi and plasma membranes in yeast via PI(4)P binding [63,64]. SopF was fused to yEGFP and expressed in wild type yeast and the six PI kinase yeast mutants (genetic deletions or Tet-off strains); localization was visualized by fluorescence microscopy of live cells (Fig 3A, S3B Fig) and protein production was monitored by immunoblotting with anti-GFP antibodies (S3C Fig). yEGFP-SopF was produced at equivalent levels in all yeast strains upon galactose induction (S3C Fig). Notably, the steady-state subcellular localization of SopF in yeast (internal membrane sites; Fig 3A, S3B Fig) and mammalian cells (plasma membrane; Figs 2C and 3B) differed, similar to what has previously been reported for some secreted bacterial proteins [58,65]. This may be a consequence of imaging in live (yeast) versus formaldehyde-fixed (HeLa) cells. Localization of yEGFP-SopF was unchanged in *fab1*Δ, *lsb6*Δ, *vps34*Δ, *pik1*^tet-off^ and *stt4*^tet-off^ yeast strains (Fig 3A, S3B Fig). However, yEGFP-SopF showed increased plasma membrane targeting and decreased punctate localization in the *mss4*^tet-off^ strain (Fig 3A, S3B Fig), revealing that the steady-state localization of SopF is dependent on the activity of Mss4. Mss4 is the major PI(4)P 5-kinase in *S*. *cerevisiae* [66,67]. It localizes to the plasma membrane where it directs PI(4,5)P_2_ synthesis and is required for the maintenance of actin cytoskeleton organization and endocytosis [66–68]. While total cellular levels of PI(4,5)P_2_ are reduced by 65% in a Mss4 mutant, total levels of PI(3)P, PI(4)P and PI(3,5)P_2_ are unaffected [66,68,69]. At the subcellular level, however, a Mss4 mutant has decreased PI(4,5)P_2_ and increased PI(4)P accumulation at the plasma membrane [69,70]. Therefore, concomitant with PI(4,5)P_2_ depletion and/or PI(4)P accumulation at the plasma membrane, SopF redistributes from punctate intracellular compartments to the plasma membrane in yeast.

To investigate the relationship between SopF localization and phosphoinositide pools in mammalian cells, we used fluorescently-tagged phospholipid biosensors and microscopy. HeLa cells were co-transfected with mCherry-SopF and either EGFP or EGFP-PH domain chimeras, then fixed and visualized by confocal microscopy (Fig 3B). Compared to EGFP alone, co-transfection with EGFP-PH-phospholipase C δ1 (PLCδ1), EGFP-2xPH-Osh2 or EGFP-PH-Bruton’s tyrosine kinase (Btk) appeared to promote the plasma membrane localization of mCherry-SopF (Fig 3B). The PH domain of PLCδ1 serves as a lipid biosensor for PI(4,5)P_2_ and localizes exclusively to the plasma membrane [71]. We found extensive co-localization of SopF and PH-PLCδ1 at the periphery of cells, particularly in filopodia (Fig 3B). PI(4)P-binding PH domain-GFP chimeras concentrate either at the plasma membrane or Golgi in mammalian cells—the PH domain of Osh2 localizes predominantly at the plasma membrane and weakly at the Golgi, whereas the PH domains of oxysterol binding protein (OSBP), four phosphate adaptor protein (FAPP1) and SidC only label PI(4)P pools at the Golgi [64,72,73]. We observed colocalization of SopF with 2xPH-Osh2 at the plasma membrane, particularly in filopodial extensions (Fig 3B), but not with Golgi-targeting PI(4)P-binding probes (PH-FAPP1 or P4C-SidC; S4A Fig). The Btk PH domain specifically binds to PI(3,4,5)P_2_ [74] and PH-Btk co-localized with mCherry-SopF staining in lamellipodia at the cell periphery (Fig 3B). Lastly, the PI(3)P binding FYVE domain from Hrs (2xFYVE-Hrs), which concentrates on early endosomes [75], did not colocalize with mCherry-SopF (S4A Fig).

The above studies suggested that SopF localization was connected to host cell phospholipids. We therefore tested whether SopF could directly bind phospholipids using a protein-lipid overlay assay. SopF was heterologously produced in *Escherichia coli* as a glutathione S-transferase (GST) fusion protein, affinity purified and used to probe nitrocellulose-immobilized phosphoinositides and lipids on PIP Strips. Bound protein was detected using anti-GST antibodies. Compared to GST alone, which displayed no binding affinity for phosphoinositides or lipids, GST-SopF bound to phosphatidic acid and a number of phosphoinositides *in vitro*, specifically PI(3)P, PI(3,5)P_2_ and PI(3,4,5)P_3_ (Fig 3C). A preference for SopF binding to PI(3,5)P_2_ was indicated by PIP Arrays (S4B Fig). Collectively, this data revealed that SopF subcellular targeting in eukaryotic cells is phosphoinositide-dependent and SopF binds phosphoinositides *in vitro*.

### SopF promotes the maintenance of nascent SCV membrane integrity

**Fig 4:**
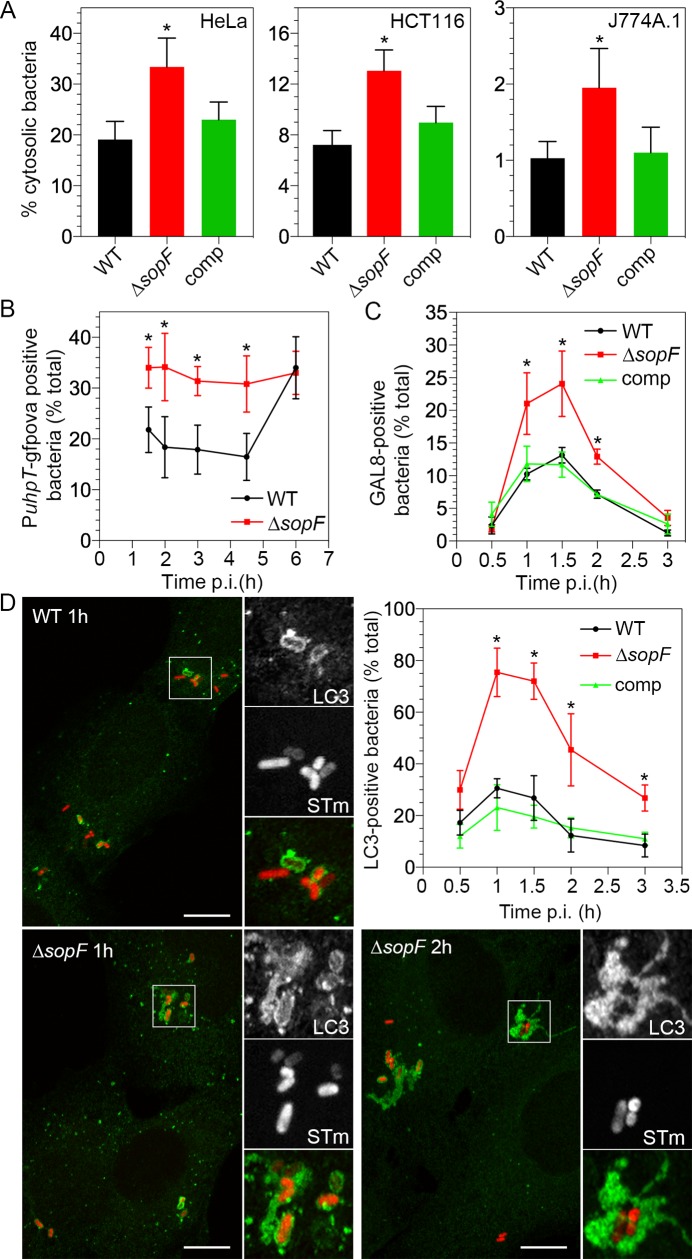
SopF promotes nascent SCV membrane integrity. **(A)** HeLa epithelial cells (left panel), HCT116 epithelial cells (middle panel) and J774A.1 mouse macrophage-like cells (right panel) were infected with *S*. Typhimurium wild type (WT), Δ*sopF* or Δ*sopF* pSopF-3xFLAG (comp) bacteria. The chloroquine resistance assay was used to quantify the proportion of cytosolic bacteria at 90 min p.i. Data represent the mean ± SD (n≥3 independent experiments). Asterisks indicate data significantly different from WT infection (one-way ANOVA with Dunnett’s post-hoc test). **(B)** Fluorescence detection of cytosolic *S*. Typhimurium. Wild type (WT) and Δ*sopF* bacteria constitutively expressing mCherry and harboring a P*uhpT*-*gfpova* reporter were used to infect HeLa cells. The unstable GFP variant (GFP-OVA) is under the control of the *S*. Typhimurium *uhpT* promoter, which is induced by glucose-6-phosphate, a metabolite found exclusively in the mammalian cytosol. GFP fluorescence is therefore indicative of *S*. Typhimurium that are in damaged vacuoles and/or free in the cytosol. The number of GFP-positive bacteria was scored by fluorescence microscopy. Data represent mean ± SD (n≥5 independent experiments). Asterisks indicate data significantly different from WT bacteria (Student’s t-test). **(C)** Timecourse of galectin-8 (GAL8) association. HeLa cells were infected with *S*. Typhimurium wild type (WT), Δ*sopF* or Δ*sopF* pSopF-3xFLAG (comp) bacteria (all strains are constitutively expressing mCherry) and at the indicated times, infected monolayers were fixed and immunostained for GAL8, which decorates damaged SCVs. The number of GAL8-positive bacteria was quantified by fluorescence microscopy. Data represent mean ± SD (total of >450 bacteria per strain from 3 independent experiments). Asterisks indicate data significantly different from WT bacteria (one-way ANOVA with Dunnett’s post-hoc test). **(D)** Timecourse of LC3 association. HeLa cells were infected as in (C) and at the indicated times cells were fixed and immunostained for microtubule-associated protein 1A/1B-light chain 3 (LC3), a marker of autophagy. The number of bacteria associated with LC3 was quantified by fluorescence microscopy. Data are the mean ± SD (n≥3 experiments). Asterisks represent data significantly different from WT bacteria (one-way ANOVA with Dunnett’s post-hoc test). Representative confocal images show LC3 association (green) with bacteria (red, STm) at 1 h p.i. and 2 h p.i. Scale bars are 10 μm. Insets are enlargements of the boxed areas.

We have shown that SopF is a T3SS1 translocated effector that targets eukaryotic cell membranes and binds phosphoinositides. While SopF is not overtly required for *S*. Typhimurium invasion into non-phagocytic cells (Fig 1C; [41]) or intracellular replication (S1 Fig), T3SS1 and its dedicated effectors also promote SCV biogenesis, trafficking and lysis. A small but significant proportion of *S*. Typhimurium lyse their internalization vacuole, but no type III effectors that modulate the frequency of this event have yet been identified. To test whether SopF is involved in vacuole maintenance, we infected HeLa epithelial cells with wild type and Δ*sopF* bacteria and monitored nascent SCV lysis using three independent assays: (i) the chloroquine (CHQ) resistance assay, (ii) a cytosolic reporter plasmid and (iii) GAL8 recruitment. CHQ selectively kills vacuolar but not cytosolic *Salmonella* and when used in combination with a gentamicin protection assay it allows for the quantification of the proportion of total bacteria that are present in the host cell cytosol [34,76]. Using the CHQ resistance assay we found that there was a significant increase in the proportion of Δ*sopF* bacteria present in the cytosol of HeLa cells compared to wild type bacteria at 90 min p.i. (Fig 4A). This phenotype was also observed in colonic epithelial cells, HCT116 (Fig 4A), and mouse macrophage-like cells, J774A.1 (Fig 4A). Complementation of Δ*sopF* bacteria with plasmid-borne SopF-3xFLAG restored the efficiency of nascent vacuole lysis to wild type levels (Fig 4A).

The P*uhpT-gfpova* plasmid has previously been used as a biosensor for *S*. Typhimurium exposure to the mammalian cytosol [77]. Expression of the unstable GFP variant, GFP-OVA, is under the control of the glucose-6-phosphate responsive *S*. Typhimurium *uhpT* promoter. Since glucose-6-phosphate is present in the mammalian cytosol and not the lumen of the SCV, GFP-OVA fluorescence is indicative of the sub-population of intracellular *S*. Typhimurium that are in damaged vacuoles and/or free in the cytosol. HeLa cells were seeded on glass coverslips and infected with *S*. Typhimurium wild type and Δ*sopF* bacteria (constitutively expressing mCherry on the chromosome) harboring the P*uhpT-gfpova* plasmid. At various times p.i., cells were fixed and the number of GFP-positive bacteria scored by fluorescence microscopy. GFP-positive bacteria were not detectable until 90 min p.i., presumably reflecting the time required for GFP-OVA to be produced, fold and fluoresce after initial bacterial exposure to the epithelial cytosol. By 90 min p.i., approximately 20% of wild type bacteria were GFP-positive and this level was maintained at a steady-state until 6 h p.i., whereupon there was a significant increase in the proportion of cytosolic wild type bacteria (Fig 4B), coincident with the initiation of rapid replication in the epithelial cytosol [33,78]. Significantly more Δ*sopF* bacteria were GFP-positive at 1.5 h, 2 h, 3 h and 4.5 h p.i. (Fig 4B), indicating that upon deletion of *sopF* there is an increased frequency of *S*. Typhimurium in damaged vacuoles and/or free in the cytosol over this timeframe. Altogether, data from these independent assays identify SopF as a T3SS1 effector that promotes the integrity of the nascent SCV membrane. Interestingly, despite an increased nascent vacuole lysis for the *sopF* deletion mutant, an equivalent proportion of wild type and Δ*sopF* bacteria was present in the epithelial cytosol at later times (≥6 h p.i.) according to the fluorescent biosensor assay (Fig 4B), CHQ resistance assay (49 ± 8.2% cytosolic bacteria for wild type and 46 ± 9.7% for the Δ*sopF* mutant at 7 h p.i. in HeLa cells, n≥6 independent experiments) and single-cell analysis (S1 Fig).

Galectins are β-galactoside-binding lectins that act as host “danger receptors” by monitoring endosomal and lysosome integrity [30]. Fluorescently-tagged galectin-3 (GAL3) has been used as a marker of vacuole lysis by *Shigella flexneri* [29,79], *Listeria monocytogenes* [29], *Legionella pneumophila* [80] and *S*. Typhimurium [16]. It localizes to the limiting membrane of damaged bacteria-containing vacuoles [16,29]. GAL8 and galectin-9 (GAL9) also decorate ruptured SCVs [30]. GAL3, GAL8 and GAL9 recruitment to damaged vacuoles is transient in nature because of their disappearance once the membrane is repaired or disassembled. We used endogenous GAL8 as a measure of vacuole integrity over a 3 h time course of infection. HeLa cells seeded on glass coverslips were infected with wild type and Δ*sopF* bacteria (constitutively expressing mCherry on the chromosome) and at various times, fixed and immunostained for GAL8. The number of GAL8-positive bacteria was scored by fluorescence microscopy. We found that the recruitment of GAL8 by wild type bacteria peaked at 90 min p.i. (13.1±1.2%, Fig 4C), in agreement with a previous study using fluorescently-tagged GAL8 [30]. Significantly more Δ*sopF* bacteria were decorated with GAL8 at 1 h, 1.5 h and 2 h p.i. (Fig 4C), indicating enhanced SCV disruption. *In trans* complementation of Δ*sopF* bacteria with SopF-3xFLAG restored the amplitude and kinetics of GAL8 acquisition to wild type levels (Fig 4C).

GAL8 directly binds NDP52 to direct the autophagy machinery to damaged SCVs [30]. Polyubiquitin coating of cytosolic S. Typhimurium is also sensed by NDP52, along with two other autophagy adaptor proteins, p62/SQSTM1 and optineurin. All three autophagy adaptors promote engulfment of *S*. Typhimurium by autophagosomes by direct binding to microtubule-associated protein 1A/1B light chain 3B (LC3) [81–84]. By immunofluorescence analysis, we found that the *sopF* deletion mutant colocalized more frequently with p62/SQSTM1 and LC3 than wild type bacteria (Fig 4D, S5 Fig). Particularly striking was robust LC3 recruitment to Δ*sopF* bacteria at 1 h and 2 h p.i. Rather than forming a discrete ring around bacteria, like for wild type *S*. Typhimurium, LC3 accumulation was cloud-like and often tubular in nature around Δ*sopF* bacteria (Fig 4D). Collectively our GAL8, p62 and LC3 recruitment data indicates that the *sopF* deletion mutant is subject to enhanced disruption of the nascent vacuole and detection and capture by the autophagy pathway.

### The C-terminus of SopF is required for its membrane association and biological activity

**Fig 5:**
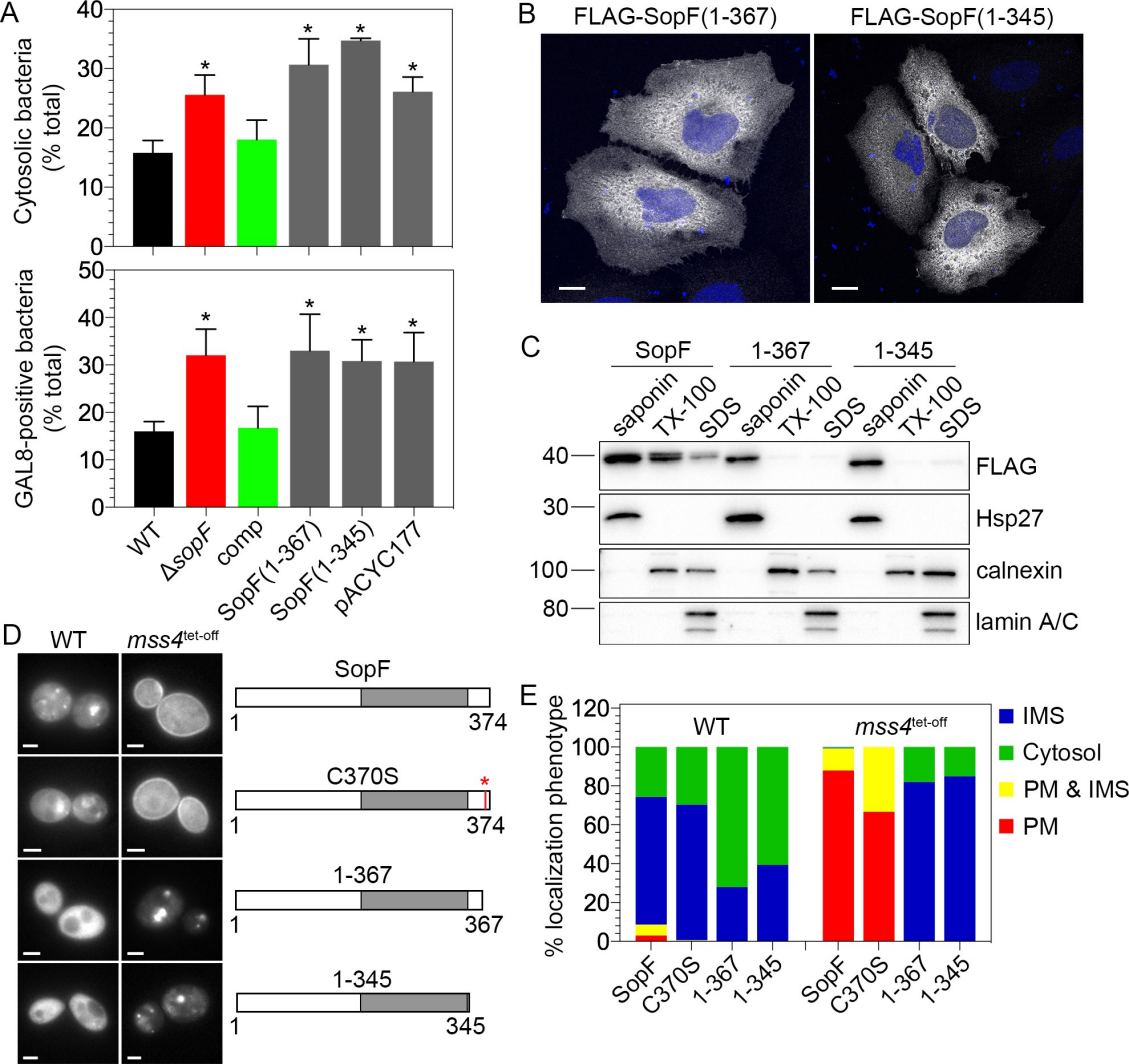
The C-terminus of SopF is required for its membrane association in eukaryotic cells. **(A)** HeLa epithelial cells were infected with *S*. Typhimurium wild type (WT), Δ*sopF*, Δ*sopF* pSopF (comp), Δ*sopF* pSopF(1–345), Δ*sopF* pSopF(1–367) or Δ*sopF* pACYC177 (empty vector) bacteria. The proportion of cytosolic bacteria was determined by CHQ resistance assay at 90 min p.i. (upper panel) or GAL8 recruitment at 1 h p.i. (lower panel, all bacteria are constitutively expressing mCherry for fluorescence detection). Upper panel: data represent the mean ± SD (n≥3 independent experiments). Lower panel: Data represent the mean ± SD (total of >600 bacteria per strain from n≥3 independent experiments). Asterisks indicate data significantly different from WT infection (one-way ANOVA with Dunnett’s post-hoc test). **(B)** HeLa cells were transfected with plasmids encoding for FLAG-SopF(1–367) or FLAG-SopF(1–345) for 18 h. Cells were fixed and immunostained with anti-FLAG antibodies. DNA was stained with Hoechst 33342. Representative confocal microscopy images show FLAG-SopF in greyscale and DNA in blue. Scale bars are 10 μm. **(C)** Subcellular fractionation of transfected cells. HeLa cells were transfected with plasmids encoding for FLAG-SopF, FLAG-SopF(1–367) or FLAG-SopF(1–345) for 18 h, then collected and subjected to sequential detergent fractionation. Equal volumes of saponin-soluble, TX-100-soluble, and SDS-soluble fractions were separated by SDS-PAGE and subject to immunoblotting with antibodies against the FLAG epitope, Hsp27 (cytosol), calnexin (membranes) and lamin A/C (nucleus). Molecular mass markers are indicated on the left. Results are representative of two independent experiments. **(D)** C-terminal truncations of SopF lose plasma membrane association in the *S*. *cerevisiae mss4*^tet-off^ strain. Wild type (WT) and *mss4*^tet-off^ yeast strains were transformed with plasmids encoding for yEGFP-SopF, yEGFP-SopF C370S, yEGFP-SopF(1–367) or yEGFP-SopF(1–345) and the subcellular localization of SopF in live cells was visualized by widefield fluorescence microscopy. Representative fluorescence images are shown. Scale bars are 2 μm. The role of a potential lipidation site in SopF localization was assessed by site-directed mutagenesis of the Cys370 residue (C370S). The grey box depicts a domain of unknown function (DUF), DUF3626, spanning amino acid residues 178–338 of SopF. **(E)** Quantification of SopF localization in WT and *mss4*^tet-off^ yeast strains that were transformed and visualized as described in (D). Subcellular localization was categorized as cytosol, internal membrane sites (IMS), plasma membrane (PM), or IMS and PM. Results are expressed as the mean percentage of total yeast transformants (n = 300 cells from three independent transformations).

SopF is a 374 amino acid protein with a predicted molecular mass of 42 kDa. It contains a domain of unknown function (DUF), DUF3626, spanning residues 178–338. Basic local alignment analyses (e.g. BLAST) of SopF do not yield any significant matches to sequences of proteins of known function, but secondary structure predictions (e.g. PHYRE2, PSIPRED) suggest that SopF has a high content of α-helices (50%). Many of these are in the N-terminus (amino acids 14–23, 31–43, 65–70, 75–116, 121–133, 144–162, 177–190). The N-terminus of type III effectors is required for their translocation [85–87], minimizing the feasibility of making N-terminal SopF deletions and retaining effector translocation. Instead we focused on the C-terminus of SopF for structure-function analysis. Based upon the location of the DUF3626 domain and the prediction by PHYRE2 [88] that amino acids 334–343 and 354–366 of SopF adopt α-helical structures, we created two SopF truncations, SopF(1–367) and SopF(1–345) and assessed whether deleting these regions affected SopF function during *S*. Typhimurium infection. HeLa cells were infected with wild type, Δ*sopF* or Δ*sopF* bacteria complemented *in trans* with SopF full-length, SopF(1–345) or SopF(1–367) and nascent SCV lysis was assessed via the CHQ resistance assay at 90 min p.i. and GAL8 recruitment at 1 h p.i. (Fig 5A). SopF(1–345) and SopF(1–367) were produced (S6A Fig) and translocated by *S*. Typhimurium (S6B Fig, S6C Fig), but neither truncation was able to restore vacuole lysis efficiency of the *sopF* mutant to wild type levels, whereas full-length SopF did (Fig 5A). From these results we can conclude that the C-terminus of SopF is required for its biological function to promote SCV membrane integrity.

We next assessed the effect of C-terminal truncations on the localization of SopF in eukaryotic cells. To do this, we ectopically expressed two SopF truncations—FLAG-SopF(1–345) or FLAG-SopF(1–367)—in HeLa cells and monitored their steady-state localization by immunofluorescence of fixed cells and sequential detergent fractionation. Unlike FLAG-SopF, which was associated with plasma membrane ruffles and filopodia (Fig 2B), the immunostaining pattern of both SopF truncations was primarily cytosolic (Fig 5B). This altered subcellular localization was confirmed by sequential detergent fractionation, where the majority of FLAG-SopF(1–345) and FLAG-SopF(1–367) was solubilized with 0.1% (w/v) saponin treatment (Fig 5C), which releases cytosolic proteins. Comparable results were obtained with EGFP-tagged SopF truncations (S2B Fig, S2C Fig). Therefore, deletion of as few as seven amino acids from the C-terminus of SopF prevents its association with mammalian cell membranes.

We also examined the impact of C-terminal truncations on the subcellular localization of SopF in *S*. *cerevisiae*. yEGFP-SopF(1–345) and yEGFP-SopF(1–367) were expressed in wild type and *mss4*^tet-off^ yeast strains; protein production was monitored by immunoblotting with anti-GFP antibodies and subcellular localization by fluorescence microscopy of live cells. Both SopF truncations were produced in yeast upon galactose induction (S3D Fig). In contrast to yEGFP-SopF, which primarily localized to internal membrane sites, pEGFP-SopF(1–345) and yEGFP-SopF(1–367) appeared predominantly cytosolic in wild type yeast (Fig 5D and 5E). Furthermore, in the *mss4*^tet-off^ mutant, while full length SopF redistributed to the plasma membrane, neither of the SopF truncations did (Fig 5D and 5E). This narrows down the Mss4-dependent localization phenotype to the carboxyl-terminal seven amino acids of SopF (368-RDCIILY-374). To further refine which residue(s) within this heptapeptide are subcellular localization determinants, we mutated individual amino acids and tested their effect on localization. First, we mutated the Cys370 residue to test whether it might be a lipidation site. The post-translational addition of lipid groups such as palmitate, farnesyl or geranylgeranyl to cysteine residues can facilitate the membrane association of proteins, including bacterial effectors [61,89]. For example, host prenylation of a C-terminal CAAX motif in the *Salmonella* type III effector, SifA, promotes its association with late endosomal/lysosomal membranes [55] and host S-palmitoylation of several *S*. Typhimurium and *L*. *pneumophila* effectors directs their membrane association upon translocation [90–92]. However, the subcellular localization of the SopF C370S mutant was indistinguishable from SopF in wild type and *mss4*^tet-off^ yeast strains (Fig 5D and 5E). Individual mutation of the remaining six amino acids (R368A, D369A, I371A, I372A, L373A and Y374A) also failed to affect the localization of SopF in wild type and *mss4*^tet-off^ yeast strains (S3E Fig, S3F Fig). Therefore, no single amino acid within this heptapeptide defines the subcellular localization of SopF in wild type or Mss4-deficient *S*. *cerevisiae*. Collectively, the results from this structure-function analysis indicate that membrane association and biological activity of SopF in eukaryotic cells are dependent on the carboxyl-terminal seven amino acids, implying that SopF must be membrane-associated to fulfil its biological function.

### SopF and SopB have opposing activities on SCV membrane integrity

**Fig 6:**
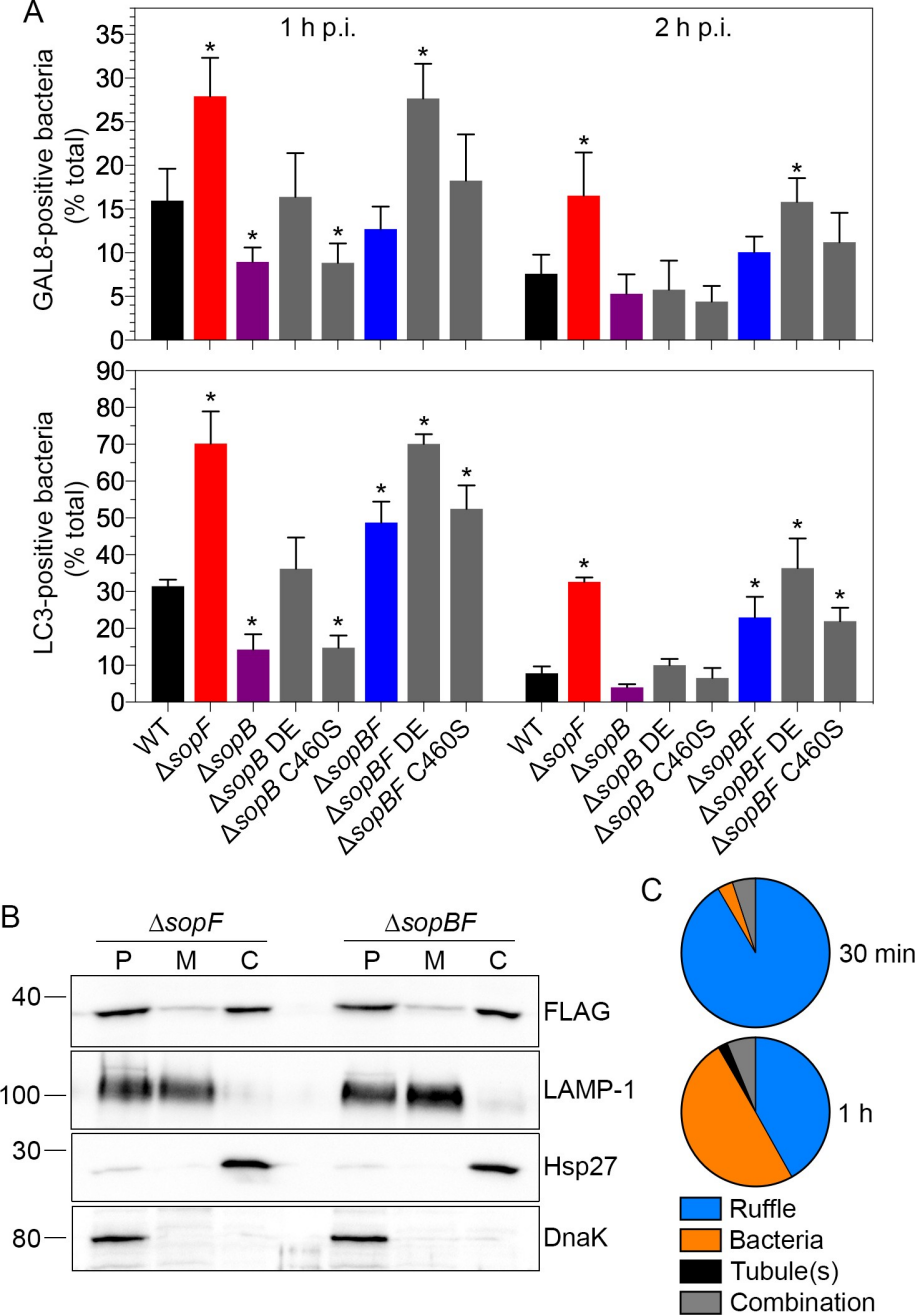
SopF and SopB have opposing effects on vacuole stability. **(A)** HeLa cells were infected with the following mCherry-expressing *S*. Typhimurium strains–wild type (WT), Δ*sopF*, Δ*sopB*, Δ*sopB* pWKSDE (*in trans* complementation with SopB/SigD and its cognate chaperone SigE, Δ*sopB* DE), Δ*sopB* pWSKDE C460S (*in trans* complementation with SopB/SigD C460S and its cognate chaperone SigE, Δ*sopB* C460S), Δ*sopB*Δ*sopF* (Δ*sopBF*), Δ*sopB*Δ*sopF* pWSKDE (Δ*sopBF* DE) or Δ*sopB*Δ*sopF* pWSKDE C460S (Δ*sopBF* C460S). Monolayers were fixed at 1 h and 2 h p.i. and immunostained for GAL8 (upper panel), a marker of damaged SCVs, or the autophagy-associated protein, LC3 (lower panel). The number of GAL8- or LC3-positive bacteria was scored by fluorescence microscopy. Data represent the mean ± SD (n≥3 independent experiments). Asterisks indicate data significantly different from WT infection for each timepoint (one-way ANOVA with Dunnett’s post-hoc test). **(B)** Translocated SopF fractionates to host cell membranes independent of SopB. HeLa cells were infected with *S*. Typhimurium Δ*sopF* pSopF-3xFLAG or Δ*sopB*Δ*sopF* (Δ*sopBF*) pSopF-3xFLAG bacteria. At 1 h p.i., cells were mechanically disrupted and subject to differential centrifugation to obtain three fractions–P (unbroken host cells, host cell nuclei, intact bacteria), M (host cell membranes) and C (host cell cytosol). Equal volumes were separated by SDS-PAGE and subject to immunoblotting with antibodies directed against FLAG, LAMP-1 (lysosomal membranes), Hsp27 (cytosol) and DnaK (bacteria). Molecular mass markers are indicated on the left. Results are representative of two independent experiments. **(C)** HeLa cells were infected with Δ*sopB*Δ*sopF* pSopF-3xFLAG bacteria and the localization of translocated SopF was determined by tyramide signal amplification followed by fluorescence microscopy. Pie charts show the mean percentage distribution of SopF localization patterns at 30 min and 1 h p.i. (n = 3 independent experiments). Categories are plasma membrane ruffles, bacteria, membrane tubule(s) originating from the SCV or a combination of these (bacteria plus ruffles, ruffles plus tubule(s) or bacteria plus tubules).

Phosphoinositides are key regulators of host-pathogen interactions, including SCV trafficking and identity [93]. The T3SS1 effector, SopB/SigD, is an inositol phosphatase that modulates the phosphoinositide composition of *Salmonella*-induced ruffles and the nascent SCV [18–20]. Given that SopB generates specific phosphoinositides during an infection and SopF binds phosphoinositides *in vitro*, along with the tendency for T3SS1 effectors to act on common cellular pathways [15,50,94,95], we examined whether there was any crosstalk between SopF and SopB biological activities. HeLa cells were infected with the following mCherry-expressing *S*. Typhimurium strains: wild type, Δ*sopF*, Δ*sopB*, Δ*sopB* pWSKDE (*in trans* complementation with SopB/SigD and SigE, its type III chaperone), Δ*sopB* pWSKDE C460S (*in trans* complementation with the catalytically inactive SopB/SigD C460S and SigE), Δ*sopB*Δ*sopF*, Δ*sopB*Δ*sopF* pWSKDE and Δ*sopB*Δ*sopF* pWSKDE C460S and nascent SCV damage (GAL8) and autophagic capture (LC3) were monitored by immunostaining of fixed cells at 1 h and 2 h p.i. (Fig 6A). Compared to wild type bacteria, more Δ*sopF* bacteria were decorated by GAL8 at 1 h and 2 h p.i., whereas significantly fewer Δ*sopB* bacteria were GAL8-positive at 1 h p.i., but not 2 h p.i. (Fig 6A, upper panel). This result suggests that SopB and SopF might have counteracting activities on membrane stabilization during early vacuole biogenesis (≤1 h p.i.). In agreement, Δ*sopB*Δ*sopF* bacteria were comparable to wild type bacteria for GAL8 acquisition at 1 h and 2 h p.i. (Fig 6A, upper panel). *In trans* complementation of Δ*sopB*Δ*sopF* bacteria with SopB (Δ*sopB*Δ*sopF* pWSKDE) restored levels of GAL8 acquisition to that observed for the *sopF* deletion mutant, confirming that the altered phenotype for the double deletion mutant was specifically due to the absence of SopB. Mutation of the Cys460 residue of SopB (C460S) abrogates its 4- and 5-phosphatase activity on phosphoinositides *in vitro* [96,97]. Providing SopB C460S (pWSKDE C460S) *in trans* failed to complement the proportion of Δ*sopB*Δ*sopF* bacteria in damaged vacuoles back to the level of Δ*sopF* bacteria, or Δ*sopB* bacteria back to wild type levels (Fig 6A, upper panel). The trend for accumulation of the autophagosomal membrane marker, LC3, mirrored that of GAL8, with the exception of the double deletion mutant (Fig 6A, lower panel). The Δ*sopB*Δ*sopF* mutant showed a significant increase in LC3 recruitment compared to wild type bacteria at 1 h and 2 h p.i., to a level intermediate that of wild type and Δ*sopF* bacteria (Fig 6A, lower panel). *In trans* complementation of Δ*sopB*Δ*sopF* bacteria with SopB, but not SopB C460S, fully restored LC3 accumulation back to Δ*sopF* levels (Fig 6A, lower panel).

Our structure-function studies (Fig 5) indicated that SopF must associate with host cell membranes in order to modulate the stability of the nascent SCV. One explanation for the different levels of vacuole damage observed for Δ*sopF* and Δ*sopB*Δ*sopF* bacteria (Fig 6A) was if translocated SopF depends on SopB’s inositol phosphatase activity for its membrane association. To test this, HeLa cells were infected with Δ*sopF* pSopF-3xFLAG and Δ*sopB*Δ*sopF* pSopF-3xFLAG bacteria and at 1 h p.i., monolayers were subject to mechanical lysis followed by differential centrifugation at 6,500 x*g* and 100,000 x*g* to obtain three fractions: P (6,500 x*g* pellet, contains host cell nuclei, intact bacteria and unbroken cells), M (100,000 x*g* pellet, contains host cell membranes) and C (100,000 x*g* supernatant, contains host cell cytosol). The content of these fractions was assessed by immunoblotting with anti-DnaK (intact bacteria), anti-Hsp27 (host cell cytosol), anti-LAMP-1 (host cell membranes) and anti-FLAG (SopF-3xFLAG) antibodies. For the Δ*sopF* pSopF-3xFLAG infection, translocated SopF-3xFLAG was detected in M and C fractions, indicating partitioning to host cell membranes and cytosol, respectively (Fig 6B). Upon infection with Δ*sopB*Δ*sopF* bacteria, SopF-3xFLAG translocation appeared comparable to Δ*sopF* bacteria, both in total amount and partitioning profile (Fig 6B). We also compared SopF localization by immunofluorescence. HeLa cells were infected with Δ*sopF* and Δ*sopB*Δ*sopF* bacteria harboring pSopF-3xFLAG (both strains constitutively expressing mCherry) and the localization of translocated SopF was examined using tyramide-coupled immunofluorescence with anti-FLAG antibodies. The number of SopF-positive cells was equivalent for Δ*sopF* and Δ*sopB*Δ*sopF* bacteria at 30 min and 1 h p.i. (23.0% and 21.6% of infected cells for Δ*sopF* bacteria at 1 h and 2 h p.i., respectively; 27.7% and 22.9% for Δ*sopB*Δ*sopF* bacteria at 1 h and 2 h p.i., respectively (mean from 3 independent experiments)). The distribution pattern of translocated SopF staining–ruffles, bacteria and/or tubules–was also comparable for Δ*sopF* (Fig 1D) and Δ*sopB*Δ*sopF* bacteria at 30 min and 1 h p.i. (Fig 6C). Altogether, from these results we can conclude that translocated SopF associates with host cell membranes independently of SopB.

## Discussion

While bacterial secretion systems are essential to the virulence of many Gram-negative pathogens, their insertion into the bacteria-containing vacuole membrane also acts as an “Achilles heel” because it damages the membrane, which allows cytosolic surveillance pathways to detect the presence of bacteria in these damaged vacuoles [31]. Bacterial pathogens that are tailored for life in a vacuole, such as *S*. Typhimurium, must possess strategies to minimize vacuole membrane damage. Exemplifying this, *S*. Typhimurium has T3SS needle tip proteins that confer relatively poor damaging/lytic ability compared to their counterparts from cytosolic bacteria [35]. Here we identify an additional mechanism used by *S*. Typhimurium to promote the integrity of its nascent SCV membrane, SopF, a type III effector that is specific to *Salmonella enterica* and *Salmonella bongori* spp. The importance of SopF and nascent vacuole integrity to the pathogenesis of *S*. Typhimurium is illustrated by results from animal models of infection. In a transposon-directed insertion-site sequencing (TraDIS) screen of *S*. Typhimurium mutants for their relative fitness during intestinal colonization of pigs, cows and chickens after oral infection, a *sopF* (*SL1344_1177*) mutant was significantly attenuated in all three animal models [98]. In the same study, a *sopF* mutant was not attenuated for systemic infection of mice upon intravenous infection [98]. However, upon oral infection of streptomycin-pretreated mice, a *sopF* mutant is outcompeted by wild type bacteria for colonization of the cecum, spleen and liver at 24 h [41]. *sopF* is therefore part of a core set of genes required for efficient intestinal colonization by *S*. Typhimurium, irrespective of the host species.

We report that SopF targets the nascent SCV to promote membrane stability. We and others have previously noted the high prevalence of bacterial effectors that associate with host cell membranes [56,58,99]. SopF does not appear to be stably associated with cell membranes, either when ectopically expressed or bacterially translocated, as only a portion of the total pool partitions to this fraction (Figs 2A, 2B and 6B). This result argues that SopF is a peripheral membrane protein, which is supported by bioinformatics analysis that predicts SopF does not have any transmembrane spanning regions (TMpred program). Our structure-function studies pinpoint that the carboxy-terminus of SopF plays a critical role in its membrane targeting in eukaryotic cells, and subsequently its biological activity (Fig 5). This region comprises only seven amino acids, with four of these being hydrophobic residues, and is predicted by PHYRE2 to adopt a β-strand structure. We predict that this heptapeptide sequence is critical for phosphoinositide and/or protein interactions that promote the membrane association of SopF. Of note, short hydrophobic stretches in YopE and ExoS, type III effectors from *Yersinia* spp. and *Pseudomonas aeruginosa*, respectively, function as membrane localization domains in eukaryotic cells [100] and some small GTPases rely on a short stretch of hydrophobic amino acids or polybasic clusters to facilitate their plasma membrane binding [101].

Purified SopF binds a number of phosphoinositides *in vitro*, hinting that membrane targeting of SopF is mediated by phosphoinositide binding *in vivo*. This does not preclude that protein-protein interactions are also involved. Alto and colleagues showed that the localization of a number of *Salmonella* effectors (PipB, PipB2, SopA, SseG, SseJ and SteA) is affected in yeast PI kinase mutants, implying that their subcellular targeting also depends on phosphoinositide-binding [58]. The authors further identified that purified recombinant PipB2, SopA and SteA bound phospholipids *in vitro* [58]. The findings for SteA corroborated an earlier study that demonstrated that this T3SS2 effector localizes to the SCV membrane via its interaction with PI(4)P [60]. Phosphoinositide-binding is not unique to *S*. Typhimurium effectors and, in fact, is quite widespread for type III and type IV effectors. DrrA/SidM [102], SidC [102–106], Lpg1101 and Lpg2603 [107] from *Legionella* spp. bind PI(4)P, LpnE, LtpD, LtpM and RidL from *L*. *pneumophila* [108–111] and CvpB from *Coxiella burnetii* [112] bind PI(3)P, and ExoU from *Pseudomonas aeruginosa* [113] and VopR from *Vibrio parahaemolyticus* [57] bind PI(4,5)P_2_ for example.

The phosphoinositide binding preference of SopF *in vivo* remains unclear. SopF preferentially binds PI(3,5)P_2_ in lipid overlay assays but we were unable to assess PI(3,5)P_2_ binding in eukaryotic cells because there is currently no known specific PI(3,5)P_2_-binding probe. PI(3,5)P_2_ is much less abundant than other phosphoinositides in mammalian cells and localizes on early endosomes, late endosomes and lysosomes [114]. Perturbation of PI(3,5)P_2_ synthesis in yeast (*fab1*Δ mutant) did not change the subcellular localization of SopF, however (Fig 3A). By contrast, its subcellular localization was affected in yeast defective for proper PI(4,5)P_2_ synthesis (*mss4*^tet-off^ strain, Fig 3A). It remains possible that the SopF localization defect in the *mss4* mutant is not specifically due to the alteration of phosphoinositide pools at the plasma membrane but rather an indirect effect of aberrant actin cytoskeleton organization, Rho1-mediated signaling or sphingolipid biosynthesis [66,67,115]. Upon ectopic expression in mammalian cells, we observed partial colocalization of SopF with multiple PH domain probes that have specificity for different phosphoinositide pools at the plasma membrane (Fig 3B). The nascent SCV is known to be decorated with PI(4)P [60] but PI(4,5)P_2_ is absent [20,116]. The direct accumulation of PI(3,5)P_2_ on the SCV has not been monitored. The association of SopF with host cell membranes is independent of SopB (Fig 6), the *S*. Typhimurium effector whose inositol phosphatase activity largely governs the phosphoinositide composition of the nascent SCV. Consequently, we propose that host-generated phosphoinositides promote the membrane recruitment of translocated SopF. Our ongoing studies are directed towards identification of which specific phosphoinositide(s) are bound by SopF during infection.

Why does only a subset of *S*. Typhimurium lyse their internalization vacuole? In an elegant study by Enninga and colleagues, it was shown that fusion, or not, of the nascent SCV (designated the *Salmonella*-containing compartment (SCC) by the authors) with macropinosomes determines the fate of *S*. Typhimurium in epithelial cells [16]. By live-cell imaging, the majority of SCCs that fused with macropinosomes remained intact whereas all bacteria that eventually hyper-replicated in the cytosol of epithelial cells had initially failed to fuse with macropinosomes. It is likely that SCC fusion with macropinosomes provides new membrane to allow for vacuole expansion. This study therefore established a direct correlation between SCC-macropinosome fusion and vacuole integrity, which is in contrast to the early events during infection with the professional cytosolic pathogen, *S*. *flexneri*. Internalized *S*. *flexneri* are surrounded by and come in contact with newly formed macropinosomes, but do not fuse with them, and then rapidly lyse their internalization vacuole [117]. There is a positive correlation between the number of macropinosomes in the vicinity of wild type *S*. *flexneri* and the timing of vacuole lysis, however. Infection with a *S*. *flexneri ipgD* mutant led to a 60% reduction in the number of macropinosomes surrounding bacteria [117] and a delay in the kinetics of vacuole rupture compared to wild type *S*. *flexneri* [79]. It has been shown that SopB, an IpgD homolog, contributes to macropinosome formation following *Salmonella* infection [15,21]. Similar to a *S*. *flexneri ipgD* mutant, here we describe that a *S*. Typhimurium *sopB* deletion mutant has a transient delay in nascent vacuole lysis compared to wild type bacteria; it is defective at 1 h p.i., but not 2 h p.i. (Fig 6A). SopB and IpgD both control the phosphoinositide composition of the vacuole membrane and might modulate bacteria-containing vacuole rupture in a similar macropinosome-dependent manner, perhaps via the recruitment of Rab11 [79,117] or sorting nexins [97,118–120].

While SopF is the first bacterial factor reported to promote nascent SCV stability, a number of host factors have been previously implicated in this process; some promote stability and some instability. TANK-binding kinase 1 (TBK1) was shown to maintain the integrity of internalization vacuoles containing Gram-negative and Gram-positive bacteria, including that of the nascent SCV [121]. TBK1 also recruits autophagy-associated proteins to promote clearance of cytosolic *S*. Typhimurium [84]. The phosphoinositide 3-phosphatase, myotubularin 4 (MTMR4), promotes SCV stability in fibroblasts. It is recruited to the nascent SCV as early as 15 min p.i. [122] and in MTMR4-depleted A431 cells, an increase in GAL8-, LC3- and p62-positive SCVs at 2 h and 3 h p.i., but not 1 h p.i., is observed [122]. Patrick et al. (2018) recently reported that recruitment of the retromer sorting complex stabilizes the nascent SCV. shRNA-mediated knockdown of VPS35, a component of the retromer sorting complex, led to an increase in the proportion of cytosolic *S*. Typhimurium at 1 h and 2 h p.i. in HeLa cells [123]. They proposed a model whereby VPS35 is initially recruited to the nascent SCV, then displaced from the SCV due to its interaction with SseC, a T3SS2 translocon protein. While the fate of Δ*sseC* bacteria was not reported in this study, this mutant is T3SS2 translocation-defective and such mutants remain in an intact SCV [124], hinting that the retromer displacement model for vacuole stabilization might be more complex than proposed. The lone host protein known to antagonize nascent SCV membrane stability is the coat protein complex II (COPII). Santos *et al*. (2015) found that COPII was recruited to the nascent SCV and siRNA mediated knockdown of a COPII component, Sec13, halved the proportion of *S*. Typhimurium in ruptured SCVs in HeLa cells at 1 h p.i. [125]. COPII recruitment therefore promotes nascent SCV rupture in epithelial cells. Using numerous techniques, we have not identified an interaction between SopF and any of these above-mentioned host proteins, suggesting that SopF is acting in a novel manner to modulate nascent SCV integrity.

Bacterial effectors can act alone, or they can interact with other effectors to modulate host functions; such effector-effector interactions can be categorized as direct or indirect [126]. “Meta-effectors” describes the scenario whereby one effector acts directly on another to modulate its function and is typified by the *L*. *pneumophila* type IV effectors LubX and SidH–LubX polyubiquitinates SidH to regulate its levels, and thus activity, inside of host cells [127]. Effectors that act cooperatively or antagonistically on a shared host target or pathway represent indirect effector-effector interactions. One example of cooperativity is the five *Salmonella* type III effectors—SipA, SipC, SopB, SopE and SopE2—that target the host cell actin cytoskeleton to promote plasma membrane ruffling and bacterial invasion. SopE and SptP from *Salmonella* are hallmark examples of effectors with antagonistic functions; SopE-mediated activation of Rho GTPases is counteracted by the GTPase-activating activity of SptP [14]. SopF and SopB have opposing effects on nascent vacuole membrane dynamics and provide a new example of antagonistic T3SS1 effectors. Interestingly, other antagonistic effector-effector relationships have been reported for the maintenance of vacuole integrity by *S*. Typhimurium (SifA/SseJ and SifA/SopD2) and *L*. *pneumophila* (SdhA/PlaA). *S*. Typhimurium has a second type III effector, SifA, that stabilizes the mature SCV membrane. SifA is translocated by T3SS2 and acts much later than SopF during the infection process; a *sifA* mutant is not defective for maintaining vacuole integrity until ≥6 h p.i. [124,128]. SifA interacts with the host protein SKIP (PLEKHM2) to downregulate the recruitment of kinesin, a molecular motor, to the SCV and affect vacuolar membrane dynamics [129,130]. SseJ is a T3SS2 effector [86] with phospholipase and acyltransferase activity [131,132]. Unlike Δ*sifA* bacteria, a *sifA sseJ* deletion mutant is not defective for stability of the mature SCV membrane [132,133] indicating that the loss of vacuolar membrane around Δ*sifA* bacteria requires SseJ. Similarly, a *sifA sopD2* deletion mutant also resides within an intact vacuole [134] highlighting that *sopD2* is epistatic over *sifA*. The biological function of SopD2 is not known. *L*. *pneumophila* utilizes the Dot/Icm secretion system to translocate type IV effectors that customize its intracellular niche. It was recently identified that SdhA and PlaA have antagonistic activities regarding stability of the *Legionella*-containing vacuole (LCV) [80]. In the absence of *sdhA*, the LCV is unstable, resulting in bacterial release into the cytosol, their autophagic capture and degradation. A suppressor screen identified *plaA* as being able to rescue the Δ*sdhA* replication defect. PlaA has phospholipase activity, like its homolog SseJ from *S*. Typhimurium. The mechanistic basis for how SdhA/PlaA, SifA/SseJ, SifA/SopD2 and SopB/SopF counteract each other’s activities remains undetermined. Regardless, the expanding number of effector proteins identified to have antagonistic activities in regard to bacteria-containing vacuole membrane dynamics reinforces that bacterial control over vacuole integrity is an important virulence strategy.

## Materials and methods

### Bacterial strains

*S*. Typhimurium SL1344 was the wild type strain used in this study [135]. The SL1344 Δ*ssaR*, Δ*prgI*::FRT and Δ*sopB* strains have been described previously [34,136,137]. An in-frame unmarked deletion of *sopF* (deletion of amino acids 3–373 of SopF) was constructed using allelic exchange in a counter-selectable suicide vector harboring SacB, pRE112, as previously described [138]. Briefly, a non-polar deletion cassette was amplified using overlap extension PCR from *S*. Typhimurium SL1344 genomic DNA (prepared using a Bactozol DNA isolation kit (Molecular Research Center, Inc.)) (oligonucleotide sequences are provided in S1 Table), ligated into pRE112 and transformed into *E*. *coli* SY327λpir, a donor strain for conjugation into SL1344 wild type and Δ*sopB* strains. Resulting meridiploids were incubated at 30°C overnight on agar containing 1% (w/v) tryptone, 0.5% (w/v) yeast extract and 5% (w/v) sucrose. Sucrose-resistant clones were screened for deletion of *sopF* by PCR with primers flanking the recombination region. The resulting strains were designated SL1344 Δ*sopF* and Δ*sopB*Δ*sopF*. SL1344 wild type *glmS*::*Ptrc*-*mCherryST* constitutively expresses mCherry (codon-optimized for *S*. Typhimurium) under the control of the *trc* promoter and has been described previously [139]. P22 lysate derived from this strain was used to transduce Δ*sopF*, Δ*sopB* and Δ*sopB*Δ*sopF* bacteria to create Δ*sopF glmS*::*Ptrc-mCherryST*, Δ*sopB glmS*::*Ptrc-mCherryST* and Δ*sopB*Δ*sopF glmS*::*Ptrc-mCherryST* strains, respectively, followed by removal of the Cm^R^ cassette using pCP20 [140]. For construction of a chromosomal 3xFLAG-tagged derivative of SopF (SL1344 *sopF*::3xFLAG), recombinational transfer of the 3xFLAG coding sequence to the 3’ end of the *sopF* coding sequence was achieved using the oligonucleotides SL1177-3xFLAG-for and SL1177-3xFLAG-rev with pSUB11 as a template, following the method described by Uzzau et al. (2001) [141].

### Plasmid DNA constructs

For detection of type III effector translocation, a fusion of SopF to adenylate cyclase (CyaA) was constructed. To create pSopF-CyaA, 417 bp upstream of the *sopF* start codon plus the genetic region encoding for amino acids (1–199) of SopF was amplified from SL1344 genomic DNA, in addition to the catalytic domain of *Bordetella pertussis* CyaA from pMS107 [142]. These two fragments were then mixed in a second round of PCR, and the resulting amplicon was digested with SmaI/XhoI and ligated into the corresponding sites of pACYC177 (New England Biolabs). The SopB(1–200)-CyaA-SigE (pACYC177 backbone) and SseK1-CyaA (pACYC184 backbone) plasmids have been described previously [48,143]. Alternatively, SopF, SopF(1–367) and SopF(1–345) fusions to TEM1 β-lactamase were constructed in pCX340 [144] to detect type III effector translocation.

For complementation of Δ*sopF* bacteria, the entire coding sequence of *sopF* and 417 bp of upstream region were amplified from SL1344 genomic DNA with Sma-SL1177CyaA-F and Xho-SL1177-R, digested with SmaI/XhoI and ligated into SmaI/XhoI-digested pACYC177 to create pSopF. The SopF truncations—pSopF(1–345) and pSopF(1–367)—were constructed similarly. For plasmid-borne expression of 3xFLAG-tagged SopF, we used the oligonucleotides Sma-SL1177CyaA-F and Xho-SL1177FLAG-R with SL1344 *sopF*::3xFLAG genomic DNA as a template. The resulting amplicon was digested with SmaI/XhoI and ligated into the corresponding sites of pACYC177 to create pSopF-3xFLAG. The pSopF-2xHA plasmid was constructed using SL1344 wild type genomic DNA as an amplification template with Sma-SL1177CyaA-F and Xho-SL11772HA-R oligonucleotides, followed by ligation into SmaI/XhoI-digested pACYC177. The SopB complementation plasmids–pWSKDE and pWSKDE C460S - are in a low copy number plasmid backbone (pWSK29, [145]) and have been described previously [22,146].

SL1344 wild type *glmS*::*Ptrc-mCherryST* and Δ*sopF glmS*::*Ptrc-mCherryST* strains harboring the *PuhpT-gfpova* plasmid [77] were used as a biosensor for bacterial exposure to the mammalian cytosol. Expression of the unstable GFP variant, GFP-OVA, is under the control of the glucose-6-phosphate responsive *S*. Typhimurium *uhpT* promoter.

For ectopic expression in mammalian cells, SopF was cloned into pFLAG-pcDNA4/TO (pFLAG; [147]), pEGFP-C2 (Clontech) or pmCherry-C1 (Clontech) to create pFLAG-SopF, pEGFP-SopF and pmCherry-SopF, respectively. C-terminal truncations of SopF were cloned into pFLAG-pcDNA4/TO and pEGFP-C2. SopF, SopF truncations and SopF amino acid point mutants were cloned into p413Gal-yEGFP (yEGFP; [58]) for galactose-inducible expression in *S*. *cerevisiae*. All constructed plasmids were verified by sequencing.

The following plasmids encoding for yEGFP- or EGFP-labeled lipid-binding domains were used as biosensors of cellular phospholipid pools: yEGFP-2xPH-Osh2 [58], EGFP-2xPH-Osh2 [64], PH-PLCδ1-EGFP [148], PH-Btk-EGFP [149], EGFP-2xFYVE-Hrs [150], P4C-SidC-EGFP [60] and PH-FAPP1-EGFP [151].

### Mammalian cell culture

HeLa (ATCC CCL-2) human cervical adenocarcinoma epithelial cells, HCT116 (ATCC CCL-247) human colorectal carcinoma epithelial cells and J774A.1 (ATCC TIB-67) mouse macrophage-like cells were purchased from American Type Culture Collection (ATCC) and used within 15 passages of receipt. Cells were maintained in the growth medium recommended by ATCC, all containing 10% (v/v) heat-inactivated fetal calf serum (FCS; Gemini Bio Products). Tissue culture plasticware was purchased from Thermo Scientific Nunc.

### Bacterial infection of mammalian cells

Mammalian cells were seeded at the following densities: (1) HeLa epithelial: 5x10^4^cells/well or 6x10^4^ cells/well (glass coverslips) in 24-well plates or 2x10^5^ cells/well in 6-well plates 24 h prior to infection; (2) HCT116 epithelial: 1x10^5^ cells/well in 24-well plates or 3.4x10^5^ cells/well in 6-well plates ~42–44 h prior to infection; (3) J774A.1 macrophage-like: 2x10^5^ cells/well or 2.5x10^5^ cells/well (glass coverslips) in 24-well plates or 8x10^5^ cells/well in 6-well plates 24 h prior to infection. Unless otherwise stated, T3SS1-induced bacterial subcultures were prepared in LB-Miller broth (Difco) [76] and HeLa (MOI ~50), HCT116 (MOI ~20) and J774A.1 (MOI ~10) cells were infected for 10 min with bacterial subcultures as described [76]. Gentamicin protection and CHQ resistance assays (400 μM CHQ for all cell lines) were as described previously [76].

### Translocation assays

To quantify type III effector-CyaA translocation, J774A.1 cells were seeded in 24-well plates the day prior to infection. *S*. Typhimurium wild type, Δ*prgI*::FRT and Δ*ssaR* strains harboring pSopF(1–199)-CyaA, pSopB(1–200)-CyaA-SigE or pSseK1-CyaA plasmids were grown as 3.5 h subcultures (to assess T3SS1-dependent translocation) or as overnight stationary phase cultures (to assess T3SS2-dependent translocation) as described [45,76]. J774A.1 cells were infected with bacterial subcultures for 10 min at an MOI of ~10 (for wild type and Δ*ssaR* strains) or ~20 (for the Δ*prgI*::FRT strain). Under these conditions, equivalent CFU were internalized for all three strains and <6% macrophage cytotoxicity was observed at 1 h p.i. as determined by CytoTox 96 Non-Radioactive Cytotoxicity Assay (Promega). Monolayers were washed with PBS at 1 h p.i., then lysed and processed for cAMP quantification as described previously [47]. Alternatively, J774A.1 cells were infected with overnight stationary phase cultures (MOI ~50) by centrifugation at 500 x*g* for 5 min (t_0_), followed by a further incubation at 37°C in 10% CO_2_ for 25 min. Cells were washed three times in Hanks’ balanced salt solution (HBSS), then incubated in growth media containing 50 μg/ml gentamicin for 1 h, followed by 10 μg/ml gentamicin until 8 h p.i., when lysates were collected and processed as described above. cAMP was measured using the Amersham cAMP Biotrak enzyme immunoassay system (GE Healthcare BioScience) according to the manufacturer’s instructions for the non-acetylation procedure.

TEM1 β-lactamase translocation assays were performed as follows. J774A.1 macrophage-like cells were seeded in tissue culture treated black 96-well plates (Cellvis for microscope imaging or Corning for fluorescence plate reader detection) at 4x10^4^ cells/well two days prior to infection. Subcultures of *S*. Typhimurium SL1344 wild type carrying pCX340, pCX340-SopF, pCX340-SopF(1–345) or pCX340-SopF(1–367) were inoculated from overnight cultures at a 1:10 dilution in LB-Miller broth. After 3.5 h growth at 37°C, shaking at 220 rpm, cultures were induced with 1 mM isopropyl β-D-1 thiogalactopyranoside (IPTG; Fisher) for 1 h. J774A.1 cells were infected with bacterial subcultures for 20 min (MOI ~20). Non-internalized bacteria were removed by washing thrice with HBSS, and cells were loaded with CCF2-AM fluorescent substrate in the presence of 2.5 mM probenecid (Biotium) and 1 mM IPTG for 90 min at room temperature according to the manufacturer’s instructions for the LiveBLAzer FRET-B/G loading kit (Thermo). Wells were then washed once in HBSS and incubated in FluoroBrite DMEM Live Cell Fluorescence Imaging Media (Gibco) containing 2.5 mM probenecid. β-lactamase activity was assessed by fluorescence microscopy or in a fluorescence plate reader. Images of live cells from randomly chosen fields were acquired as a single 1 μm section on a Leica SP8 confocal microscope upon excitation at 405 nm and separate collection of fluorescence emissions with either a 420–480 nm (blue fluorescence) or a 520–560 nm bandpass (green fluorescence). Images were assembled using Adobe Photoshop CS6. Alternatively, fluorescence was quantified on a TECAN SPARK plate reader with excitation at 410 nm (20 nm bandpass), and emission was detected via 450 nm (20 nm band pass, blue fluorescence) and 520 nm (20 nm band pass, green fluorescence) filters. Translocation was expressed as the emission ratio at 450/520 nm and normalized to mock-infected cells for each experiment.

### Transient transfection of mammalian cells

Plasmid DNA was purified using the Nucleobond Xtra Midi Plus kit (Macherey-Nagel) according to the manufacturer’s protocol. HeLa cells were transfected with plasmid DNA using the FuGENE 6 transfection reagent (Promega) according to the manufacturer’s protocol. Cells were transfected for 16–18 h with 500 ng DNA/well (24-well plate) or 1 μg DNA/well (6-well plate).

### Fractionation of mammalian cells

For analysis of the membrane association of SopF when ectopically expressed, transfected HeLa cells were subject to sequential detergent fractionation as described previously [23,56], with minor modifications. HeLa cells were seeded in 6-well plates at 1.4–1.8 x 10^5^ cells/well and transfected for 18–20 h prior to fractionation. Transfected cells were sequentially treated with 0.1% (w/v) saponin, 0.5% (v/v) TX-100, then 2.5% (w/v) SDS (resuspension of the final pellet in 1.5x SDS-PAGE sample buffer). Equal volumes of saponin-, TX-100- and SDS-soluble fractions were analyzed by immunoblotting.

The subcellular association of translocated SopF was determined by mechanical lysis of infected HeLa cells followed by differential centrifugation as previously described [56,152], with minor modifications. HeLa cells were seeded in 10 cm dishes at 1.5 x 10^6^ cells/dish the day prior to infection. Bacterial subcultures of Δ*sopF* pSopF-3xFLAG and Δ*sopB*Δ*sopF* pSopF-3xFLAG bacteria were used to infect monolayers for 10 min (MOI ~50) (two dishes per strain). At 1 h p.i., cells were mechanically disrupted by 4–5 passes through a 22-gauge needle, followed by low-speed centrifugation at 6,500 x*g* for 10 min to pellet nuclei, unbroken cells and intact bacteria. The supernatant was further subject to a high-speed centrifugation at 100,000 x*g* for 30 min to separate host cell membranes from cytosol. Equal volumes of each fraction were subject to immunoblotting with anti-FLAG, anti-Hsp27 (cytosol), anti-LAMP-1 (membranes) and anti-DnaK (intact bacteria) antibodies.

### Immunofluorescence microscopy

HeLa cells were seeded on acid-washed, 12 mm glass coverslips (#1.5 thickness, Fisher Scientific) in 24-well plates. Transfected or infected cells were fixed for 10 min at 37°C with 2.5% (w/v) paraformaldehyde (PFA; EMD Millipore). Fixed cells were washed three times with PBS then permeabilized-blocked in PBS containing 10% normal goat serum (NGS; Gibco) and 0.2% (w/v) saponin (Acros) for 20 min. Primary and secondary antibodies were diluted in blocking buffer. Coverslips were incubated with the following primary antibodies for 45 min at room temperature: mouse anti-FLAG M2 affinity isolated (1:500 dilution; Sigma), rabbit anti-VASP (clone 9A2, 1:100; Cell Signaling), rabbit anti-moesin (clone Q480, 1:100; Cell Signaling), rabbit anti-lamellipodin (clone D8A2K, 1:100, Cell Signaling), mouse anti-human galectin-8 (clone 210608, 1:100; R&D Systems), guinea pig polyclonal anti-p62 (1:200; Progen), rabbit polyclonal anti-LC3 (1:300; MBL). Coverslips were washed three times with PBS and incubated for a further 30–45 min at room temperature with Alexa Fluor-conjugated secondary antibodies (1:400 dilution; Life Technologies) in blocking buffer. After three washes in PBS, cells were incubated with Hoechst 33342 (1:10,000; Invitrogen) for 1 min before mounting in Mowiol on glass slides. Samples were visualized using a Leica DM4000 upright fluorescence microscope. Image acquisition was on a Leica SP8 Scanning Point confocal microscope using the sequential acquisition mode through an optical section of 0.3 μm in the z-axis. Images are maximum intensity projections of z-stacks.

To detect translocated SopF, tyramide signal amplification for immunofluorescent enhancement was used. HeLa cells seeded on glass coverslips were infected with Δ*sopF glmS*::*Ptrc-mCherryST* or Δ*sopB*Δ*sopF glmS*::*Ptrc-mCherryST* bacteria harboring pSopF-3xFLAG. At 30 min and 1 h p.i., monolayers were fixed and blocked/permeabilized as described above. The subsequent staining procedure followed the manufacturer’s instructions for the Alexa Fluor 488 Tyramide SuperBoost kit (Invitrogen). Primary antibody—mouse anti-FLAG M2 affinity isolated (1:2,000 dilution; Sigma)—and poly-HRP-conjugated secondary antibody incubations were for 45 min at room temperature. The tyramide amplification step was for 5 min at room temperature.

### Immunoblotting

Proteins were separated by SDS-PAGE and transferred to 0.2 μm or 0.45 μm pore-size nitrocellulose membranes (GE Healthcare Life Sciences). Membranes were blocked at room temperature for 1 h with Tris-buffered saline (TBS) containing 5% (w/v) skim milk powder and 0.1% (v/v) Tween-20 (TBST-milk), then incubated with the following primary antibodies overnight at 4˚C: mouse anti-FLAG M2 affinity isolated (1:2,000 dilution; Sigma), mouse anti-HA.11 ascites (1:2,000; BioLegend), rabbit polyclonal anti-GFP (1:40,000; Thermo), mouse anti-β-lactamase (clone 8A5.A10, 1:2,000 dilution; Thermo), mouse anti-Hsp27 (clone G31, 1:20,000; Cell Signaling), rabbit polyclonal anti-calnexin (1:40,000; Enzo), rabbit polyclonal anti-lamin A/C (1:5,000; Cell Signaling) or mouse anti-LAMP-1 (clone H4A3, 1:1,000 dilution; Developmental Studies Hybridoma Bank). The hybridoma H4A3 developed by J.T. August and J.E.K. Hildreth (The Johns Hopkins University School of Medicine) was obtained from the Developmental Studies Hybridoma Bank, created by the NICHD of the NIH and maintained at The University of Iowa, Department of Biology, Iowa City, IA 52242. Blots were then incubated with anti-rabbit IgG or anti-mouse IgG horseradish peroxidase (HRP)-conjugated secondary antibodies (1:10,000; Cell Signaling) in TBST-milk for 1–2 h at room temperature, followed by Supersignal West Femto Max Sensitivity ECL Substrate (Thermo). Chemiluminescence was detected using a ChemiDoc MP Imaging System (Bio-Rad) and Bio-Rad Image Lab software.

### Recombinant protein production and purification

*E*. *coli* Rosetta 2(DE3) (Novagen) harboring pGEX-6P-1 and pGEX-6P-1-SopF plasmids were used for recombinant protein production. Overnight bacterial cultures were subcultured to logarithmic phase (OD_600_ of 0.6–0.8) and GST fusion production was induced overnight at 18˚C by the addition of 1 mM IPTG (AppliChem). Bacteria were harvested by centrifugation at 10,000 x*g* for 15 minutes at 4˚C. All subsequent steps were performed on ice or at 4˚C. The cell pellet was resuspended in Tris-buffered saline (TBS) (50 mM Tris-HCl pH 8.0, 150 mM NaCl) containing cOmplete EDTA-free protease inhibitor cocktail (Roche), then lysed using an Avestin EmulsiFlex-C3 high-pressure cell homogenizer or a Constant Systems Ltd. CF cell disruptor. Bacterial lysates were centrifuged at 13,000 x*g* for 30 min, after which the clarified supernatant was loaded into a PolyPrep Chromatography Column (Bio-Rad) pre-packed with equilibrated glutathione sepharose beads (Merck). Beads were then washed once with TBS and bound protein was eluted with TBS containing 10 mM glutathione (Sigma). Eluted protein was dialyzed overnight against TBS and purified protein was aliquoted and stored at -80˚C.

### Protein-lipid overlay assays

PIP Strips and PIP Arrays (Echelon Biosciences) were used to test lipid binding of SopF according to the manufacturer’s protocol. Briefly, PIP Strips and PIP Arrays were blocked in PBS/0.1% (v/v) Tween-20 (PBST) containing 1% (w/v) skim milk powder (PBST-milk) for 1 h at room temperature. Purified recombinant GST fusion protein was incubated with PIP Strips and PIP Arrays at a concentration of 1 μg/ml in PBST containing 3% (w/v) bovine serum albumin (PBST-BSA) for 1 h at room temperature. PIP Strips and PIP Arrays were washed 3 times with PBST, then probed with rabbit anti-GST antibody (1:2,000; Cell Signaling) in PBST-BSA for 1 h at room temperature. PIP Strips and PIP Arrays were washed 3 times with PBST, then incubated with anti-rabbit HRP-conjugated secondary antibodies (1:3,000; Perkin Elmer) in PBST-milk at room temperature for 1 h. Chemiluminescence signal was detected using Clarity Western ECL Substrate (Bio-Rad) and an Amersham Imager 600 machine.

### SopF localization in yeast

The following *S*. *cerevisiae* strains were used: wild type with genetic background BY4742 (*MAT*a *his3*Δ*1 leu2*Δ*0 lys2*Δ*0 ura3*Δ*0*); *lsb6*Δ, *vps34*Δ and *fab1*Δ with genetic background BY4741 (*MAT*a *his3*Δ*1 leu2*Δ*0 met15*Δ*0 ura3*Δ*0*); *stt4*^tet-off^, *pik1*^tet-off^ and *mss4*^tet-off^ with genetic background R1158 (*MAT*a *his3Δ1 leu2Δ0 met15Δ0 URA3*::*CMVp-tTA*). Competent yeast cells were transformed with p413Gal-yEGFP-SopF variants (expression is under the control of a galactose-inducible promoter) using the standard lithium acetate transformation protocol [153]. Transformants were grown for 2–3 days on minimal media agar lacking histidine. Tet-off strains were repressed with doxycycline for 24 h prior to the induction of protein expression in galactose-containing media as described previously [58]. A Leica DM4000 upright fluorescence microscope was used for visualization and image acquisition of yEGFP-tagged protein localization in live yeast.

### Statistical analysis

All experiments were conducted on at least three separate occasions, unless otherwise indicated, and results are presented as mean ± SD. Statistical analyses were performed using one-way analysis of variance (ANOVA) with Dunnett’s post-hoc test or Student’s t-test (GraphPad Prism). A p-value of ≤0.05 was considered significant.

## Supporting information

S1 Fig Δ*sopF* bacteria are not replication-deficient in epithelial cells or macrophages. HeLa epithelial cells, HCT116 epithelial cells and J774A.1 macrophage-like cells were infected with *S.* Typhimurium wild type (WT) or Δ*sopF* bacteria. Bacterial replication was monitored by gentamicin protection assay (left panels) or fluorescence microscopy (right panels, mCherry expressing bacteria). Left panels, mean ± SD, n≥3 independent experiments; right panels, each dot represents one infected cell, data is combined from two independent experiments.
(TIF)

S2 Fig Localization of ectopically expressed EGFP-SopF in mammalian cells. **(A)** EGFP-SopF partially colocalizes with actin-binding proteins found at cell adhesion sites. HeLa cells were transfected with EGFP-SopF for 18 h, then fixed and immunostained with anti-moesin, anti-lamellipodin and anti-vasodilator-stimulated phosphoprotein (VASP) antibodies. Representative confocal microscopy images show EGFP-SopF in green and moesin, lamellipodin or VASP in red. Scale bars are 10 μm. Insets show enlargements of boxed areas. **(B)** Membrane association depends on the carboxy-terminus of SopF. HeLa cells were transfected with plasmids encoding for EGFP-SopF, EGFP-SopF(1–367) or EGFP-SopF(1–345) for 18 h, then cells were collected and subject to sequential detergent fractionation. Equal volumes of saponin-soluble, TX-100-soluble and SDS-soluble fractions were separated by SDS-PAGE and subject to immunoblotting with antibodies against GFP, Hsp27 (cytosol), calnexin (membranes) and lamin A/C (nucleus). Molecular mass markers are indicated on the left. Results are representative of two independent experiments. **(C)** HeLa cells were transfected with plasmids encoding for EGFP-SopF(1–367) or EGFP-SopF(1–345) for 18 h. Cells were fixed and DNA was stained with Hoechst 33342. Representative confocal microscopy images show EGFP-SopF in greyscale and DNA in blue. Scale bars are 10 μm.
(PDF)

S3 Fig Loss of function PI kinase screen in *S. cerevisiae*. **(A)** Phosphoinositide synthesis in yeast is governed by six PI kinases (depicted in red). **(B)** The indicated yeast strains were transformed with yEGFP-SopF and its subcellular localization was visualized by widefield fluorescence microscopy and categorized as cytosol, internal membrane sites (IMS), plasma membrane (PM), or IMS and PM. Results are expressed as the mean percentage of total yeast transformants (n ≥ 200 cells from three independent transformations). **(C, D, E)** Immunoblot analysis of yEGFP-SopF production in wild type and PI kinase mutant yeast strains. Lysates were prepared from cells grown in the absence (-) or presence (+) of galactose (Gal). Proteins were subject to immunoblotting with anti-GFP antibodies. Molecular mass markers are indicated on the left. Results are representative of two independent experiments. **(F)** Wild type (WT) and *mss4*^tet-off^ yeast strains were transformed with plasmids encoding for yEGFP-SopF and the indicated yEGFP-SopF point mutants. The subcellular localization of SopF in live cells was visualized by widefield fluorescence microscopy. Representative fluorescence images are shown. Scale bars are 2 μm.
(TIF)

S4 Fig The relationship between SopF and phosphoinositides. **(A)** SopF does not colocalize with phosphoinositide pools present on early endosomes or the Golgi. HeLa cells were co-transfected with mCherry-SopF and EGFP-phosphoinositide-binding domain chimeras for 16 h and fixed. Representative confocal microscopy images show phosphoinositide-binding probes in green and mCherry-SopF in red. PI(3)P on early endosomes is bound by 2xFYVE-Hrs and PI(4)P at the Golgi is bound by P4C-SidC and PH-FAPP1. Scale bars are 10 μm. **(B)** Protein-lipid overlay assay with SopF. Recombinant GST-SopF was purified by affinity chromatography and incubated with a PIP Array (Echelon Biosciences) at 1 μg/ml. Bound protein was detected using anti-GST antibodies followed by chemiluminescence detection. The following compounds are spotted on the nitrocellulose membrane in decreasing concentrations (100 pmol to 1.56 lipid per spot): phosphatidylinositol phosphate (PI); PI(3)P; PI(4)P; PI(5)P; PI(3,4)P_2_; PI(3,5)P_2_; PI(4,5)P_2_; PI(3,4,5)P_3_.
(TIF)

S5 Fig Enhanced p62/SQSTM1 recruitment to Δ*sopF* bacteria. HeLa cells were infected with the following mCherry-expressing *S*. Typhimurium strains–wild type (WT), Δ*sopF* and Δ*sopF* pSopF-3xFLAG (comp). At the indicated times, cells were fixed and immunostained for the autophagy adaptor protein, p62/SQSTM1. The number of p62-positive bacteria was quantified by fluorescence microscopy. Data are the mean ± SD (n ≥ 3 experiments). Asterisks represent data significantly different to WT infection (one-way ANOVA with Dunnett’s post-hoc test).
(TIF)

S6 Fig C-terminal truncations of SopF are translocated into host cells. **(A)** Detection of TEM1 fusion proteins. Whole cell lysates from *S*. Typhimurium wild type harboring pCX340 (TEM1), pCX340-SopF (SopF-TEM1), pCX340-SopF(1–345) (SopF(1–345)-TEM1) or pCX340-SopF(1–367) (SopF(1–367)-TEM1) were subject to immunoblotting with antibodies against TEM1 β-lactamase and DnaK (loading control). Cultures were induced with 1 mM IPTG for 1 h prior to collection. Molecular mass markers are indicated on the left. **(B)** β-lactamase activity in infected cells. J774A.1 macrophage-like cells were infected with the *S*. Typhimurium strains indicated in (A), then cells were loaded with CCF2-AM substrate. At 2 h p.i., β-lactamase activity was detected by measuring cleavage of the CCF2-AM substrate on a fluorescence plate reader. Data is presented as the emission ratio between blue fluorescence (450 nm) and green fluorescence (520 nm). Ratios were normalized to that of mock infected cells in each experiment. Data are mean ± SD (3 independent experiments). Asterisks indicate data significantly different to TEM1 (one-way ANOVA with Dunnett’s post-hoc test). **(C)** Fluorescence microscopy detection of β-lactamase activity. J774A.1 cells were infected and loaded with CCF2-AM substrate as in (B). Blue fluorescence indicates CCF2-AM cleaved upon effector translocation, whereas uncleaved CCF2-AM emits a green fluorescence. Representative confocal images are shown. Scale bars are 20 μm.
(TIF)

S1 Table Oligonucleotides used for cloning. (DOCX)

## References

[1] Garcia-del Portillo F, Foster JW, Maguire ME, Finlay BB. Characterization of the micro-environment of *Salmonella typhimurium*-containing vacuoles within MDCK epithelial cells. Mol Microbiol. 1992;6: 3289–3297. 1484485 10.1111/j.1365-2958.1992.tb02197.x

[2] Drecktrah D, Knodler LA, Howe D, Steele-Mortimer O. *Salmonella* trafficking is defined by continuous dynamic interactions with the endolysosomal system. Traffic. 2007;8: 212–225. 10.1111/j.1600-0854.2006.00529.x 17233756 PMC2063589

[3] McGhie EJ, Brawn LC, Hume PJ, Humphreys D, Koronakis V. *Salmonella* takes control: effector-driven manipulation of the host. Curr Opin Microbiol. 2009;12: 117–124. 10.1016/j.mib.2008.12.001 19157959 PMC2647982

[4] LaRock DL, Chaudhary A, Miller SI. Salmonellae interactions with host processes. Nat Rev Microbiol. 2015;13: 191–205. 10.1038/nrmicro3420 25749450 PMC5074537

[5] Jennings E, Thurston TLM, Holden DW. *Salmonella* SPI-2 Type III Secretion System Effectors: Molecular Mechanisms And Physiological Consequences. Cell Host Microbe. 2017;22: 217–231. 10.1016/j.chom.2017.07.009 28799907

[6] Hume PJ, Singh V, Davidson AC, Koronakis V. Swiss Army Pathogen: The *Salmonella* Entry Toolkit. Front Cell Infect Microbiol. 2017;7: 348 10.3389/fcimb.2017.00348 28848711 PMC5552672

[7] Steele-Mortimer O, Brumell JH, Knodler LA, Méresse S, Lopez A, Finlay BB. The invasion-associated type III secretion system of *Salmonella enterica* serovar Typhimurium is necessary for intracellular proliferation and vacuole biogenesis in epithelial cells. Cell Microbiol. 2002;4: 43–54. 11856172 10.1046/j.1462-5822.2002.00170.x

[8] Cirillo DM, Valdivia RH, Monack DM, Falkow S. Macrophage-dependent induction of the *Salmonella* pathogenicity island 2 type III secretion system and its role in intracellular survival. Mol Microbiol. 1998;30: 175–188. 9786194 10.1046/j.1365-2958.1998.01048.x

[9] Hensel M, Shea JE, Waterman SR, Mundy R, Nikolaus T, Banks G, et al Genes encoding putative effector proteins of the type III secretion system of *Salmonella* pathogenicity island 2 are required for bacterial virulence and proliferation in macrophages. Mol Microbiol. 1998;30: 163–174. 9786193 10.1046/j.1365-2958.1998.01047.x

[10] Zhou D, Galán J. *Salmonella* entry into host cells: the work in concert of type III secreted effector proteins. Microbes Infect. 2001;3: 1293–1298. 11755417 10.1016/s1286-4579(01)01489-7

[11] Jones BD, Paterson HF, Hall A, Falkow S. *Salmonella typhimurium* induces membrane ruffling by a growth factor-receptor-independent mechanism. Proc Natl Acad Sci USA. 1993;90: 10390–10394. 10.1073/pnas.90.21.10390 8234304 PMC47780

[12] Francis CL, Ryan TA, Jones BD, Smith SJ, Falkow S. Ruffles induced by *Salmonella* and other stimuli direct macropinocytosis of bacteria. Nature. 1993;364: 639–642. 10.1038/364639a0 8350922

[13] Raffatellu M, Wilson RP, Chessa D, Andrews-Polymenis H, Tran QT, Lawhon S, et al SipA, SopA, SopB, SopD, and SopE2 contribute to *Salmonella enterica* serotype typhimurium invasion of epithelial cells. Infect Immun. 2005;73: 146–154. 10.1128/IAI.73.1.146-154.2005 15618149 PMC538951

[14] Fu Y, Galán JE. A *Salmonella* protein antagonizes Rac-1 and Cdc42 to mediate host-cell recovery after bacterial invasion. Nature. 1999;401: 293–297. 10.1038/45829 10499590

[15] Bakowski MA, Cirulis JT, Brown NF, Finlay BB, Brumell JH. SopD acts cooperatively with SopB during *Salmonella enterica* serovar Typhimurium invasion. Cell Microbiol. 2007;9: 2839–2855. 10.1111/j.1462-5822.2007.01000.x 17696999

[16] Fredlund J, Santos JC, Stévenin V, Weiner A, Latour-Lambert P, Rechav K, et al The entry of *Salmonella* in a distinct tight compartment revealed at high temporal and ultrastructural resolution. Cell Microbiol. 2018;20 10.1111/cmi.12816 29250873

[17] Mukherjee K, Parashuraman S, Raje M, Mukhopadhyay A. SopE acts as an Rab5-specific nucleotide exchange factor and recruits non-prenylated Rab5 on *Salmonella-*containing phagosomes to promote fusion with early endosomes. J Biol Chem. 2001;276: 23607–23615. 10.1074/jbc.M101034200 11316807

[18] Mallo GV, Espina M, Smith AC, Terebiznik MR, Alemán A, Finlay BB, et al SopB promotes phosphatidylinositol 3-phosphate formation on *Salmonella* vacuoles by recruiting Rab5 and Vps34. J Cell Biol. 2008;182: 741–752. 10.1083/jcb.200804131 18725540 PMC2518712

[19] Dai S, Zhang Y, Weimbs T, Yaffe MB, Zhou D. Bacteria-generated PtdIns(3)P recruits VAMP8 to facilitate phagocytosis. Traffic. 2007;8: 1365–1374. 10.1111/j.1600-0854.2007.00613.x 17645435

[20] Bakowski MA, Braun V, Lam GY, Yeung T, Heo WD, Meyer T, et al The phosphoinositide phosphatase SopB manipulates membrane surface charge and trafficking of the *Salmonella*-containing vacuole. Cell Host Microbe. 2010;7: 453–462. 10.1016/j.chom.2010.05.011 20542249

[21] Hernandez LD, Hueffer K, Wenk MR, Galán JE. *Salmonella* modulates vesicular traffic by altering phosphoinositide metabolism. Science. 2004;304: 1805–1807. 10.1126/science.1098188 15205533

[22] Knodler LA, Winfree S, Drecktrah D, Ireland R, Steele-Mortimer O. Ubiquitination of the bacterial inositol phosphatase, SopB, regulates its biological activity at the plasma membrane. Cell Microbiol. 2009;11: 1652–1670. 10.1111/j.1462-5822.2009.01356.x 19614667 PMC2762020

[23] Marcus SL, Knodler LA, Finlay BB. *Salmonella enterica* serovar Typhimurium effector SigD/SopB is membrane-associated and ubiquitinated inside host cells. Cell Microbiol. 2002;4: 435–446. 12102689 10.1046/j.1462-5822.2002.00202.x

[24] Brawn LC, Hayward RD, Koronakis V. *Salmonella* SPI1 effector SipA persists after entry and cooperates with a SPI2 effector to regulate phagosome maturation and intracellular replication. Cell Host Microbe. 2007;1: 63–75. 10.1016/j.chom.2007.02.001 18005682 PMC1885946

[25] Patel JC, Hueffer K, Lam TT, Galán JE. Diversification of a *Salmonella* virulence protein function by ubiquitin-dependent differential localization. Cell. 2009;137: 283–294. 10.1016/j.cell.2009.01.056 19379694 PMC2673707

[26] Vonaesch P, Sellin ME, Cardini S, Singh V, Barthel M, Hardt W-D. The *Salmonella* Typhimurium effector protein SopE transiently localizes to the early SCV and contributes to intracellular replication. Cellular Microbiology. 2014;16: 1723–1735. 10.1111/cmi.12333 25052734

[27] Finn CE, Chong A, Cooper KG, Starr T, Steele-Mortimer O. A second wave of *Salmonella* T3SS1 activity prolongs the lifespan of infected epithelial cells. PLoS Pathog. 2017;13: e1006354 10.1371/journal.ppat.1006354 28426838 PMC5413073

[28] Klein JA, Grenz JR, Slauch JM, Knodler LA. Controlled Activity of the *Salmonella* Invasion-Associated Injectisome Reveals Its Intracellular Role in the Cytosolic Population. MBio. 2017;8 10.1128/mBio.01931-17 29208746 PMC5717391

[29] Paz I, Sachse M, Dupont N, Mounier J, Cederfur C, Enninga J, et al Galectin-3, a marker for vacuole lysis by invasive pathogens. Cell Microbiol. 2010;12: 530–544. 10.1111/j.1462-5822.2009.01415.x 19951367

[30] Thurston TLM, Wandel MP, von Muhlinen N, Foeglein A, Randow F. Galectin 8 targets damaged vesicles for autophagy to defend cells against bacterial invasion. Nature. 2012;482: 414–418. 10.1038/nature10744 22246324 PMC3343631

[31] Feeley EM, Pilla-Moffett DM, Zwack EE, Piro AS, Finethy R, Kolb JP, et al Galectin-3 directs antimicrobial guanylate binding proteins to vacuoles furnished with bacterial secretion systems. Proc Natl Acad Sci USA. 2017;114: E1698–E1706. 10.1073/pnas.1615771114 28193861 PMC5338555

[32] Birmingham CL, Smith AC, Bakowski MA, Yoshimori T, Brumell JH. Autophagy controls *Salmonella* infection in response to damage to the *Salmonella*-containing vacuole. J Biol Chem. 2006;281: 11374–11383. 10.1074/jbc.M509157200 16495224

[33] Knodler LA, Vallance BA, Celli J, Winfree S, Hansen B, Montero M, et al Dissemination of invasive *Salmonella* via bacterial-induced extrusion of mucosal epithelia. Proc Natl Acad Sci USA. 2010;107: 17733–17738. 10.1073/pnas.1006098107 20876119 PMC2955089

[34] Knodler LA, Nair V, Steele-Mortimer O. Quantitative assessment of cytosolic *Salmonella* in epithelial cells. PLoS ONE. 2014;9: e84681 10.1371/journal.pone.0084681 24400108 PMC3882239

[35] Du J, Reeves AZ, Klein JA, Twedt DJ, Knodler LA, Lesser CF. The type III secretion system apparatus determines the intracellular niche of bacterial pathogens. Proc Natl Acad Sci USA. 2016;113: 4794–4799. 10.1073/pnas.1520699113 27078095 PMC4855615

[36] Thurston TLM, Matthews SA, Jennings E, Alix E, Shao F, Shenoy AR, et al Growth inhibition of cytosolic *Salmonella* by caspase-1 and caspase-11 precedes host cell death. Nat Commun. 2016;7: 13292 10.1038/ncomms13292 27808091 PMC5097160

[37] Kreibich S, Emmenlauer M, Fredlund J, Rämö P, Münz C, Dehio C, et al Autophagy proteins promote repair of endosomal membranes damaged by the *Salmonella* type three secretion system 1. Cell Host Microbe. 2015;18: 527–537. 10.1016/j.chom.2015.10.015 26567507

[38] Yu HB, Croxen MA, Marchiando AM, Ferreira RBR, Cadwell K, Foster LJ, et al Autophagy facilitates *Salmonella* replication in HeLa cells. MBio. 2014;5: e00865–00814. 10.1128/mBio.00865-14 24618251 PMC3952155

[39] Colgan AM, Kröger C, Diard M, Hardt W-D, Puente JL, Sivasankaran SK, et al The Impact of 18 Ancestral and Horizontally-Acquired Regulatory Proteins upon the Transcriptome and sRNA Landscape of *Salmonella enterica* serovar Typhimurium. PLoS Genet. 2016;12: e1006258 10.1371/journal.pgen.1006258 27564394 PMC5001712

[40] Smith C, Stringer AM, Mao C, Palumbo MJ, Wade JT. Mapping the Regulatory Network for *Salmonella enterica* Serovar Typhimurium Invasion. MBio. 2016;7 10.1128/mBio.01024-16 27601571 PMC5013294

[41] Cheng S, Wang L, Liu Q, Qi L, Yu K, Wang Z, et al Identification of a Novel *Salmonella* Type III Effector by Quantitative Secretome Profiling. Mol Cell Proteomics. 2017;16: 2219–2228. 10.1074/mcp.RA117.000230 28887382 PMC5724182

[42] Sabbagh SC, Forest CG, Lepage C, Leclerc J-M, Daigle F. So similar, yet so different: uncovering distinctive features in the genomes of *Salmonella enterica* serovars Typhimurium and Typhi. FEMS Microbiol Lett. 2010;305: 1–13. 10.1111/j.1574-6968.2010.01904.x 20146749

[43] den Bakker HC, Moreno Switt AI, Govoni G, Cummings CA, Ranieri ML, Degoricija L, et al Genome sequencing reveals diversification of virulence factor content and possible host adaptation in distinct subpopulations of *Salmonella enterica*. BMC Genomics. 2011;12: 425 10.1186/1471-2164-12-425 21859443 PMC3176500

[44] Desai PT, Porwollik S, Long F, Cheng P, Wollam A, Clifton SW, et al Evolutionary Genomics of *Salmonella enterica* Subspecies. mBio. 2013;4: e00579–12. 10.1128/mBio.00579-12 23462113 PMC3604774

[45] Geddes K, Worley M, Niemann G, Heffron F. Identification of new secreted effectors in *Salmonella enterica* serovar Typhimurium. Infect Immun. 2005;73: 6260–6271. 10.1128/IAI.73.10.6260-6271.2005 16177297 PMC1230965

[46] Jones MA, Wood MW, Mullan PB, Watson PR, Wallis TS, Galyov EE. Secreted effector proteins of *Salmonella dublin* act in concert to induce enteritis. Infection and Immunity. 1998;66: 5799–5804. 9826357 10.1128/iai.66.12.5799-5804.1998 PMC108733

[47] Klein JA, Dave BM, Raphenya AR, McArthur AG, Knodler LA. Functional relatedness in the Inv/Mxi-Spa type III secretion system family. Mol Microbiol. 2017;103: 973–991. 10.1111/mmi.13602 27997726

[48] Kujat Choy SL, Boyle EC, Gal-Mor O, Goode DL, Valdez Y, Vallance BA, et al SseK1 and SseK2 are novel translocated proteins of *Salmonella enterica* serovar typhimurium. Infect Immun. 2004;72: 5115–5125. 10.1128/IAI.72.9.5115-5125.2004 15322005 PMC517430

[49] Drecktrah D, Knodler LA, Galbraith K, Steele-Mortimer O. The *Salmonella* SPI1 effector SopB stimulates nitric oxide production long after invasion. Cell Microbiol. 2005;7: 105–113. 10.1111/j.1462-5822.2004.00436.x 15617527

[50] Zhou D, Chen LM, Hernandez L, Shears SB, Galán JE. A *Salmonella* inositol polyphosphatase acts in conjunction with other bacterial effectors to promote host cell actin cytoskeleton rearrangements and bacterial internalization. Mol Microbiol. 2001;39: 248–259. 11136447 10.1046/j.1365-2958.2001.02230.x

[51] Sato N, Funayama N, Nagafuchi A, Yonemura S, Tsukita S, Tsukita S. A gene family consisting of ezrin, radixin and moesin. Its specific localization at actin filament/plasma membrane association sites. J Cell Sci. 1992;103 (Pt 1): 131–143. 1429901 10.1242/jcs.103.1.131

[52] Barzik M, McClain LM, Gupton SL, Gertler FB. Ena/VASP regulates mDia2-initiated filopodial length, dynamics, and function. Mol Biol Cell. 2014;25: 2604–2619. 10.1091/mbc.E14-02-0712 24989797 PMC4148250

[53] Krause M, Leslie JD, Stewart M, Lafuente EM, Valderrama F, Jagannathan R, et al Lamellipodin, an Ena/VASP ligand, is implicated in the regulation of lamellipodial dynamics. Dev Cell. 2004;7: 571–583. 10.1016/j.devcel.2004.07.024 15469845

[54] Price CTD, Jones SC, Amundson KE, Kwaik YA. Host-mediated post-translational prenylation of novel dot/icm-translocated effectors of *Legionella pneumophila*. Front Microbiol. 2010;1: 131 10.3389/fmicb.2010.00131 21687755 PMC3109360

[55] Reinicke AT, Hutchinson JL, Magee AI, Mastroeni P, Trowsdale J, Kelly AP. A *Salmonella* typhimurium effector protein SifA is modified by host cell prenylation and S-acylation machinery. J Biol Chem. 2005;280: 14620–14627. 10.1074/jbc.M500076200 15710609

[56] Knodler LA, Ibarra JA, Pérez-Rueda E, Yip CK, Steele-Mortimer O. Coiled-coil domains enhance the membrane association of *Salmonella* type III effectors. Cell Microbiol. 2011;13: 1497–1517. 10.1111/j.1462-5822.2011.01635.x 21679290 PMC3418822

[57] Salomon D, Guo Y, Kinch LN, Grishin NV, Gardner KH, Orth K. Effectors of animal and plant pathogens use a common domain to bind host phosphoinositides. Nat Commun. 2013;4: 2973 10.1038/ncomms3973 24346350 PMC4981085

[58] Weigele BA, Orchard RC, Jimenez A, Cox GW, Alto NM. A systematic exploration of the interactions between bacterial effector proteins and host cell membranes. Nat Commun. 2017;8: 532 10.1038/s41467-017-00700-7 28912547 PMC5599653

[59] Haneburger I, Hilbi H. Phosphoinositide lipids and the *Legionella* pathogen vacuole. Curr Top Microbiol Immunol. 2013;376: 155–173. 10.1007/82_2013_341 23918172

[60] Domingues L, Ismail A, Charro N, Rodríguez-Escudero I, Holden DW, Molina M, et al The *Salmonella* effector SteA binds phosphatidylinositol 4-phosphate for subcellular targeting within host cells. Cell Microbiol. 2016;18: 949–969. 10.1111/cmi.12558 26676327

[61] Hicks SW, Galán JE. Exploitation of eukaryotic subcellular targeting mechanisms by bacterial effectors. Nat Rev Microbiol. 2013;11: 316–326. 10.1038/nrmicro3009 23588250 PMC3859125

[62] Balla T. Phosphoinositides: tiny lipids with giant impact on cell regulation. Physiol Rev. 2013;93: 1019–1137. 10.1152/physrev.00028.2012 23899561 PMC3962547

[63] Roy A, Levine TP. Multiple pools of phosphatidylinositol 4-phosphate detected using the pleckstrin homology domain of Osh2p. J Biol Chem. 2004;279: 44683–44689. 10.1074/jbc.M401583200 15271978

[64] Balla A, Kim YJ, Varnai P, Szentpetery Z, Knight Z, Shokat KM, et al Maintenance of hormone-sensitive phosphoinositide pools in the plasma membrane requires phosphatidylinositol 4-kinase IIIalpha. Mol Biol Cell. 2008;19: 711–721. 10.1091/mbc.E07-07-0713 18077555 PMC2230591

[65] Stamm CE, Pasko BL, Chaisavaneeyakorn S, Franco LH, Nair VR, Weigele BA, et al Screening *Mycobacterium tuberculosis* secreted proteins identifies Mpt64 as a eukaryotic membrane-binding bacterial effector. mSphere. 2019;4: e00354–19. 10.1128/mSphere.00354-19 31167949 PMC6553557

[66] Desrivières S, Cooke FT, Parker PJ, Hall MN. MSS4, a phosphatidylinositol-4-phosphate 5-Kinase required for organization of the actin cytoskeleton in *Saccharomyces cerevisiae*. J Biol Chem. 1998;273: 15787–15793. 10.1074/jbc.273.25.15787 9624178

[67] Homma K, Terui S, Minemura M, Qadota H, Anraku Y, Kanaho Y, et al Phosphatidylinositol-4-phosphate 5-Kinase localized on the plasma membrane is essential for yeast cell morphogenesis. J Biol Chem. 1998;273: 15779–15786. 10.1074/jbc.273.25.15779 9624177

[68] Audhya A, Emr SD. Stt4 PI 4-Kinase localizes to the plasma membrane and functions in the Pkc1-mediated MAP kinase cascade. Developmental Cell. 2002;2: 593–605. 10.1016/S1534-5807(02)00168-5 12015967

[69] Stefan CJ, Audhya A, Emr SD. The yeast synaptojanin-like proteins control the cellular distribution of phosphatidylinositol (4,5)-bisphosphate. Mol Biol Cell. 2002;13: 542–557. 10.1091/mbc.01-10-0476 11854411 PMC65648

[70] Yamamoto W, Wada S, Nagano M, Aoshima K, Siekhaus DE, Toshima JY, et al Distinct roles for plasma membrane PtdIns(4)P and PtdIns(4,5)P2 during receptor-mediated endocytosis in yeast. J Cell Sci. 2018;131 10.1242/jcs.207696 29192062

[71] Várnai P, Balla T. Visualization of phosphoinositides that bind pleckstrin homology domains: Calcium- and agonist-induced dynamic changes and relationship to Myo-[3H]inositol-labeled phosphoinositide pools. The Journal of Cell Biology. 1998;143: 501–510. 10.1083/jcb.143.2.501 9786958 PMC2132833

[72] Hammond GRV, Machner MP, Balla T. A novel probe for phosphatidylinositol 4-phosphate reveals multiple pools beyond the Golgi. The Journal of Cell Biology. 2014;205: 113–126. 10.1083/jcb.201312072 24711504 PMC3987136

[73] Dickson EJ, Jensen JB, Hille B. Golgi and plasma membrane pools of PI(4)P contribute to plasma membrane PI(4,5)P2 and maintenance of KCNQ2/3 ion channel current. PNAS. 2014;111: E2281–E2290. 10.1073/pnas.1407133111 24843134 PMC4050574

[74] Salim K, Bottomley MJ, Querfurth E, Zvelebil MJ, Gout I, Scaife R, et al Distinct specificity in the recognition of phosphoinositides by the pleckstrin homology domains of dynamin and Bruton’s tyrosine kinase. EMBO J. 1996;15: 6241–6250. 8947047 PMC452447

[75] Gillooly DJ, Morrow IC, Lindsay M, Gould R, Bryant NJ, Gaullier J-M, et al Localization of phosphatidylinositol 3‐phosphate in yeast and mammalian cells. The EMBO Journal. 2000;19: 4577–4588. 10.1093/emboj/19.17.4577 10970851 PMC302054

[76] Klein JA, Powers TR, Knodler LA. Measurement of *Salmonella enterica* internalization and vacuole lysis in epithelial cells. Methods Mol Biol. 2017;1519: 285–296. 10.1007/978-1-4939-6581-6_19 27815887

[77] Spinnenhirn V, Farhan H, Basler M, Aichem A, Canaan A, Groettrup M. The ubiquitin-like modifier FAT10 decorates autophagy-targeted *Salmonella* and contributes to *Salmonella* resistance in mice. J Cell Sci. 2014;127: 4883–4893. 10.1242/jcs.152371 25271057

[78] Malik-Kale P, Winfree S, Steele-Mortimer O. The bimodal lifestyle of intracellular *Salmonella* in epithelial cells: replication in the cytosol obscures defects in vacuolar replication. PLoS ONE. 2012;7: e38732 10.1371/journal.pone.0038732 22719929 PMC3374820

[79] Mellouk N, Weiner A, Aulner N, Schmitt C, Elbaum M, Shorte SL, et al *Shigella* subverts the host recycling compartment to rupture its vacuole. Cell Host & Microbe. 2014;16: 517–530. 10.1016/j.chom.2014.09.005 25299335

[80] Creasey EA, Isberg RR. The protein SdhA maintains the integrity of the *Legionella*-containing vacuole. Proc Natl Acad Sci USA. 2012;109: 3481–3486. 10.1073/pnas.1121286109 22308473 PMC3295292

[81] Pankiv S, Clausen TH, Lamark T, Brech A, Bruun J-A, Outzen H, et al p62/SQSTM1 Binds Directly to Atg8/LC3 to Facilitate Degradation of Ubiquitinated Protein Aggregates by Autophagy. J Biol Chem. 2007;282: 24131–24145. 10.1074/jbc.M702824200 17580304

[82] Thurston TLM, Ryzhakov G, Bloor S, von Muhlinen N, Randow F. The TBK1 adaptor and autophagy receptor NDP52 restricts the proliferation of ubiquitin-coated bacteria. Nat Immunol. 2009;10: 1215–1221. 10.1038/ni.1800 19820708

[83] Zheng YT, Shahnazari S, Brech A, Lamark T, Johansen T, Brumell JH. The adaptor protein p62/SQSTM1 targets invading bacteria to the autophagy pathway. J Immunol. 2009;183: 5909–5916. 10.4049/jimmunol.0900441 19812211

[84] Wild P, Farhan H, McEwan DG, Wagner S, Rogov VV, Brady NR, et al Phosphorylation of the Autophagy Receptor Optineurin Restricts *Salmonella* Growth. Science. 2011;333: 228–233. 10.1126/science.1205405 21617041 PMC3714538

[85] Miao EA, Scherer CA, Tsolis RM, Kingsley RA, Adams LG, Bäumler AJ, et al *Salmonella* typhimurium leucine-rich repeat proteins are targeted to the SPI1 and SPI2 type III secretion systems. Mol Microbiol. 1999;34: 850–864. 10564523 10.1046/j.1365-2958.1999.01651.x

[86] Miao EA, Miller SI. A conserved amino acid sequence directing intracellular type III secretion by *Salmonella typhimurium*. Proc Natl Acad Sci USA. 2000;97: 7539–7544. 10.1073/pnas.97.13.7539 10861017 PMC16581

[87] Knodler LA, Celli J, Hardt W-D, Vallance BA, Yip C, Finlay BB. *Salmonella* effectors within a single pathogenicity island are differentially expressed and translocated by separate type III secretion systems. Mol Microbiol. 2002;43: 1089–1103. 11918798 10.1046/j.1365-2958.2002.02820.x

[88] Kelley LA, Mezulis S, Yates CM, Wass MN, Sternberg MJE. The Phyre2 web portal for protein modeling, prediction and analysis. Nat Protoc. 2015;10: 845–858. 10.1038/nprot.2015.053 25950237 PMC5298202

[89] Roberts PJ, Mitin N, Keller PJ, Chenette EJ, Madigan JP, Currin RO, et al Rho Family GTPase modification and dependence on CAAX motif-signaled posttranslational modification. J Biol Chem. 2008;283: 25150–25163. 10.1074/jbc.M800882200 18614539 PMC2533093

[90] Hicks SW, Charron G, Hang HC, Galán JE. Subcellular targeting of *Salmonella* virulence proteins by host-mediated S-palmitoylation. Cell Host Microbe. 2011;10: 9–20. 10.1016/j.chom.2011.06.003 21767808 PMC4326042

[91] Schroeder GN, Aurass P, Oates CV, Tate EW, Hartland EL, Flieger A, et al *Legionella pneumophila* Effector LpdA Is a Palmitoylated Phospholipase D Virulence Factor. Camilli A, editor. Infection and Immunity. 2015;83: 3989–4002. 10.1128/IAI.00785-15 26216420 PMC4567653

[92] Lin Y-H, Doms AG, Cheng E, Kim B, Evans TR, Machner MP. Host cell-catalyzed S-palmitoylation mediates Golgi targeting of the *Legionella* ubiquitin ligase GobX. J Biol Chem. 2015;290: 25766–25781. 10.1074/jbc.M115.637397 26316537 PMC4646218

[93] Kerr MC, Castro NA, Karunaratne S, Teasdale RD. The Phosphoinositides: Key regulators of *Salmonella* containing vacuole (SCV) trafficking and identity. *Salmonella*—Distribution, Adaptation, Control Measures and Molecular Technologies. 2012; doi:10.5772/30761

[94] McGhie EJ, Hayward RD, Koronakis V. Cooperation between actin-binding proteins of invasive *Salmonella*: SipA potentiates SipC nucleation and bundling of actin. EMBO J. 2001;20: 2131–2139. 10.1093/emboj/20.9.2131 11331579 PMC125241

[95] Kubori T, Galán JE. Temporal regulation of *Salmonella* virulence effector function by proteasome-dependent protein degradation. Cell. 2003;115: 333–342. 10.1016/s0092-8674(03)00849-3 14636560

[96] Marcus SL, Wenk MR, Steele-Mortimer O, Finlay BB. A synaptojanin-homologous region of *Salmonella typhimurium* SigD is essential for inositol phosphatase activity and Akt activation. FEBS Lett. 2001;494: 201–207. 10.1016/s0014-5793(01)02356-0 11311241

[97] Piscatelli HL, Li M, Zhou D. Dual 4- and 5-phosphatase activities regulate SopB-dependent phosphoinositide dynamics to promote bacterial entry. Cell Microbiol. 2016;18: 705–719. 10.1111/cmi.12542 26537021

[98] Chaudhuri RR, Morgan E, Peters SE, Pleasance SJ, Hudson DL, Davies HM, et al Comprehensive assignment of roles for *Salmonella typhimurium* genes in intestinal colonization of food-producing animals. PLoS Genet. 2013;9: e1003456 10.1371/journal.pgen.1003456 23637626 PMC3630085

[99] Ivanov SS, Charron G, Hang HC, Roy CR. Lipidation by the host prenyltransferase machinery facilitates membrane localization of *Legionella pneumophila* effector proteins. Journal of Biological Chemistry. 2010;285: 34686–34698. 10.1074/jbc.M110.170746 20813839 PMC2966084

[100] Krall R, Zhang Y, Barbieri JT. Intracellular membrane localization of *Pseudomonas* ExoS and *Yersinia* YopE in mammalian cells. J Biol Chem. 2004;279: 2747–2753. 10.1074/jbc.M301963200 14597627

[101] Heo WD, Inoue T, Park WS, Kim ML, Park BO, Wandless TJ, et al PI(3,4,5)P3 and PI(4,5)P2 lipids target proteins with polybasic clusters to the plasma membrane. Science. 2006;314: 1458–1461. 10.1126/science.1134389 17095657 PMC3579512

[102] Brombacher E, Urwyler S, Ragaz C, Weber SS, Kami K, Overduin M, et al Rab1 guanine nucleotide exchange factor SidM is a major phosphatidylinositol 4-phosphate-binding effector protein of *Legionella pneumophila*. J Biol Chem. 2009;284: 4846–4856. 10.1074/jbc.M807505200 19095644 PMC2643517

[103] Weber SS, Ragaz C, Reus K, Nyfeler Y, Hilbi H. *Legionella pneumophila* exploits PI(4)P to anchor secreted effector proteins to thereplicative vacuole. PLOS Pathogens. 2006;2: e46 10.1371/journal.ppat.0020046 16710455 PMC1463015

[104] Ragaz C, Pietsch H, Urwyler S, Tiaden A, Weber SS, Hilbi H. The *Legionella pneumophila* phosphatidylinositol-4 phosphate-binding type IV substrate SidC recruits endoplasmic reticulum vesicles to a replication-permissive vacuole. Cell Microbiol. 2008;10: 2416–2433. 10.1111/j.1462-5822.2008.01219.x 18673369

[105] Dolinsky S, Haneburger I, Cichy A, Hannemann M, Itzen A, Hilbi H. The *Legionella longbeachae* Icm/Dot substrate SidC selectively binds phosphatidylinositol 4-phosphate with nanomolar affinity and promotes pathogen vacuole-endoplasmic reticulum interactions. Infect Immun. 2014;82: 4021–4033. 10.1128/IAI.01685-14 25024371 PMC4187854

[106] Luo X, Wasilko DJ, Liu Y, Sun J, Wu X, Luo Z-Q, et al Structure of the *Legionella virulence* factor, SidC reveals a unique PI(4)P-specific binding domain essential for its targeting to the bacterial phagosome. PLoS Pathog. 2015;11: e1004965 10.1371/journal.ppat.1004965 26067986 PMC4467491

[107] Hubber A, Arasaki K, Nakatsu F, Hardiman C, Lambright D, Camilli PD, et al The machinery at endoplasmic reticulum-plasma membrane contact sites contributes to spatial regulation of multiple *Legionella* effector proteins. PLoS Pathogens. 2014;10: e1004222 10.1371/journal.ppat.1004222 24992562 PMC4081824

[108] Weber SS, Ragaz C, Hilbi H. The inositol polyphosphate 5-phosphatase OCRL1 restricts intracellular growth of *Legionella*, localizes to the replicative vacuole and binds to the bacterial effector LpnE. Cellular Microbiology. 2009;11: 442–460. 10.1111/j.1462-5822.2008.01266.x 19021631

[109] Finsel I, Ragaz C, Hoffmann C, Harrison CF, Weber S, van Rahden VA, et al The *Legionella* effector RidL inhibits retrograde trafficking to promote intracellular replication. Cell Host & Microbe. 2013;14: 38–50. 10.1016/j.chom.2013.06.001 23870312

[110] Harding CR, Mattheis C, Mousnier A, Oates CV, Hartland EL, Frankel G, et al LtpD is a novel *Legionella pneumophila* effector that binds phosphatidylinositol 3-phosphate and inositol monophosphatase IMPA1. Infect Immun. 2013;81: 4261–4270. 10.1128/IAI.01054-13 24002062 PMC3811825

[111] Levanova N, Mattheis C, Carson D, To K-N, Jank T, Frankel G, et al The *Legionella* effector LtpM is a new type of phosphoinositide-activated glucosyltransferase. J Biol Chem. 2018; 10.1074/jbc.RA118.005952 30573678 PMC6393602

[112] Martinez E, Allombert J, Cantet F, Lakhani A, Yandrapalli N, Neyret A, et al *Coxiella burnetii* effector CvpB modulates phosphoinositide metabolism for optimal vacuole development. Proc Natl Acad Sci USA. 2016;113: E3260–E3269. 10.1073/pnas.1522811113 27226300 PMC4988616

[113] Tyson GH, Halavaty AS, Kim H, Geissler B, Agard M, Satchell KJ, et al A novel phosphatidylinositol 4,5-bisphosphate binding domain mediates plasma membrane localization of ExoU and other patatin-like phospholipases. J Biol Chem. 2015;290: 2919–2937. 10.1074/jbc.M114.611251 25505182 PMC4317026

[114] McCartney AJ, Zhang Y, Weisman LS. Phosphatidylinositol 3,5-bisphosphate: low abundance, high significance. Bioessays. 2014;36: 52–64. 10.1002/bies.201300012 24323921 PMC3906640

[115] Tabuchi M, Audhya A, Parsons AB, Boone C, Emr SD. The Phosphatidylinositol 4,5-Biphosphate and TORC2 Binding Proteins Slm1 and Slm2 Function in Sphingolipid Regulation. Molecular and Cellular Biology. 2006;26: 5861–5875. 10.1128/MCB.02403-05 16847337 PMC1592763

[116] Terebiznik MR, Vieira OV, Marcus SL, Slade A, Yip CM, Trimble WS, et al Elimination of host cell PtdIns(4,5)P(2) by bacterial SigD promotes membrane fission during invasion by *Salmonella*. Nat Cell Biol. 2002;4: 766–773. 10.1038/ncb854 12360287

[117] Weiner A, Mellouk N, Lopez-Montero N, Chang Y-Y, Souque C, Schmitt C, et al Macropinosomes are key players in early Shigella invasion and vacuolar escape in epithelial cells. PLoS Pathog. 2016;12: e1005602 10.1371/journal.ppat.1005602 27182929 PMC4868309

[118] Bujny MV, Ewels PA, Humphrey S, Attar N, Jepson MA, Cullen PJ. Sorting nexin-1 defines an early phase of *Salmonella*-containing vacuole-remodeling during *Salmonella* infection. J Cell Sci. 2008;121: 2027–2036. 10.1242/jcs.018432 18505799

[119] Braun V, Wong A, Landekic M, Hong WJ, Grinstein S, Brumell JH. Sorting nexin 3 (SNX3) is a component of a tubular endosomal network induced by Salmonella and involved in maturation of the *Salmonella*-containing vacuole. Cell Microbiol. 2010;12: 1352–1367. 10.1111/j.1462-5822.2010.01476.x 20482551

[120] Liebl D, Qi X, Zhe Y, Barnett TC, Teasdale RD. SopB-mediated recruitment of SNX18 facilitates *Salmonella* Typhimurium internalization by the host cell. Front Cell Infect Microbiol. 2017;7: 257 10.3389/fcimb.2017.00257 28664153 PMC5471308

[121] Radtke AL, Delbridge LM, Balachandran S, Barber GN, O’Riordan MXD. TBK1 protects vacuolar integrity during intracellular bacterial infection. PLoS Pathog. 2007;3: e29 10.1371/journal.ppat.0030029 17335348 PMC1808071

[122] Teo WX, Kerr MC, Teasdale RD. MTMR4 Is required for the stability of the *Salmonella*-containing vacuole. Front Cell Infect Microbiol. 2016;6: 91 10.3389/fcimb.2016.00091 27625994 PMC5003867

[123] Patrick KL, Wojcechowskyj JA, Bell SL, Riba MN, Jing T, Talmage S, et al Quantitative yeast genetic interaction profiling of bacterial effector proteins uncovers a role for the human retromer in *Salmonella* infection. Cell Syst. 2018;7: 323–338.e6. 10.1016/j.cels.2018.06.010 30077634 PMC6160342

[124] Beuzón CR, Méresse S, Unsworth KE, Ruíz-Albert J, Garvis S, Waterman SR, et al *Salmonella* maintains the integrity of its intracellular vacuole through the action of SifA. EMBO J. 2000;19: 3235–3249. 10.1093/emboj/19.13.3235 10880437 PMC313946

[125] Santos JC, Duchateau M, Fredlund J, Weiner A, Mallet A, Schmitt C, et al The COPII complex and lysosomal VAMP7 determine intracellular *Salmonella* localization and growth. Cell Microbiol. 2015;17: 1699–1720. 10.1111/cmi.12475 26084942

[126] Huett A. Combinatorial actions of bacterial effectors revealed by exploiting genetic tools in yeast. Mol Syst Biol. 2017;13: 911 10.15252/msb.20167447 28137776 PMC5293158

[127] Kubori T, Shinzawa N, Kanuka H, Nagai H. *Legionella* metaeffector exploits host proteasome to temporally regulate cognate effector. PLoS Pathog. 2010;6: e1001216 10.1371/journal.ppat.1001216 21151961 PMC2996335

[128] Beuzón CR, Salcedo SP, Holden DW. Growth and killing of a *Salmonella enterica* serovar Typhimurium *sifA* mutant strain in the cytosol of different host cell lines. Microbiology (Reading, Engl). 2002;148: 2705–2715. 10.1099/00221287-148-9-2705 12213917

[129] Boucrot E, Henry T, Borg J-P, Gorvel J-P, Méresse S. The intracellular fate of *Salmonella* depends on the recruitment of kinesin. Science. 2005;308: 1174–1178. 10.1126/science.1110225 15905402

[130] Dumont A, Boucrot E, Drevensek S, Daire V, Gorvel J-P, Poüs C, et al SKIP, the host target of the *Salmonella* virulence factor SifA, promotes kinesin-1-dependent vacuolar membrane exchanges. Traffic. 2010;11: 899–911. 10.1111/j.1600-0854.2010.01069.x 20406420

[131] Lossi NS, Rolhion N, Magee AI, Boyle C, Holden DW. The *Salmonella* SPI-2 effector SseJ exhibits eukaryotic activator-dependent phospholipase A and glycerophospholipid: cholesterol acyltransferase activity. Microbiology (Reading, Engl). 2008;154: 2680–2688. 10.1099/mic.0.2008/019075-0 18757801 PMC2885629

[132] Ohlson MB, Fluhr K, Birmingham CL, Brumell JH, Miller SI. SseJ deacylase activity by *Salmonella enterica* serovar Typhimurium promotes virulence in mice. Infect Immun. 2005;73: 6249–6259. 10.1128/IAI.73.10.6249-6259.2005 16177296 PMC1230951

[133] Ruiz-Albert J, Yu X-J, Beuzón CR, Blakey AN, Galyov EE, Holden DW. Complementary activities of SseJ and SifA regulate dynamics of the *Salmonella typhimurium* vacuolar membrane. Mol Microbiol. 2002;44: 645–661. 11994148 10.1046/j.1365-2958.2002.02912.x

[134] Schroeder N, Henry T, de Chastellier C, Zhao W, Guilhon A-A, Gorvel J-P, et al The virulence protein SopD2 regulates membrane dynamics of *Salmonella*-containing vacuoles. PLoS Pathog. 2010;6: e1001002 10.1371/journal.ppat.1001002 20664790 PMC2904799

[135] Hoiseth SK, Stocker BA. Aromatic-dependent Salmonella typhimurium are non-virulent and effective as live vaccines. Nature. 1981;291: 238–239. 10.1038/291238a0 7015147

[136] Brumell JH, Rosenberger CM, Gotto GT, Marcus SL, Finlay BB. SifA permits survival and replication of *Salmonella typhimurium* in murine macrophages. Cell Microbiol. 2001;3: 75–84. 11207622 10.1046/j.1462-5822.2001.00087.x

[137] Steele-Mortimer O, Knodler LA, Marcus SL, Scheid MP, Goh B, Pfeifer CG, et al Activation of Akt/protein kinase B in epithelial cells by the *Salmonella typhimurium* effector sigD. J Biol Chem. 2000;275: 37718–37724. 10.1074/jbc.M008187200 10978351

[138] Edwards RA, Keller LH, Schifferli DM. Improved allelic exchange vectors and their use to analyze 987P fimbria gene expression. Gene. 1998;207: 149–157. 10.1016/s0378-1119(97)00619-7 9511756

[139] Knodler LA, Crowley SM, Sham HP, Yang H, Wrande M, Ma C, et al Noncanonical inflammasome activation of caspase-4/caspase-11 mediates epithelial defenses against enteric bacterial pathogens. Cell Host Microbe. 2014;16: 249–256. 10.1016/j.chom.2014.07.002 25121752 PMC4157630

[140] Datsenko KA, Wanner BL. One-step inactivation of chromosomal genes in *Escherichia coli* K-12 using PCR products. Proc Natl Acad Sci USA. 2000;97: 6640–6645. 10.1073/pnas.120163297 10829079 PMC18686

[141] Uzzau S, Figueroa-Bossi N, Rubino S, Bossi L. Epitope tagging of chromosomal genes in *Salmonella*. Proc Natl Acad Sci USA. 2001;98: 15264–15269. 10.1073/pnas.261348198 11742086 PMC65018

[142] Sory MP, Cornelis GR. Translocation of a hybrid YopE-adenylate cyclase from *Yersinia enterocolitica* into HeLa cells. Mol Microbiol. 1994;14: 583–594. 7885236 10.1111/j.1365-2958.1994.tb02191.x

[143] Knodler LA, Bertero M, Yip C, Strynadka NCJ, Steele-Mortimer O. Structure-based mutagenesis of SigE verifies the importance of hydrophobic and electrostatic residues in type III chaperone function. Mol Microbiol. 2006;62: 928–940. 10.1111/j.1365-2958.2006.05418.x 17038123

[144] Charpentier X, Oswald E. Identification of the secretion and translocation domain of the enteropathogenic and enterohemorrhagic *Escherichia coli* effector Cif, using TEM-1 beta-lactamase as a new fluorescence-based reporter. J Bacteriol. 2004;186: 5486–5495. 10.1128/JB.186.16.5486-5495.2004 15292151 PMC490934

[145] Wang RF, Kushner SR. Construction of versatile low-copy-number vectors for cloning, sequencing and gene expression in *Escherichia coli*. Gene. 1991;100: 195–199. 2055470

[146] Jolly C, Winfree S, Hansen B, Steele-Mortimer O. The Annexin A2/p11 complex is required for efficient invasion of *Salmonella* Typhimurium in epithelial cells. Cell Microbiol. 2014;16: 64–77. 10.1111/cmi.12180 23931152 PMC3921270

[147] Carey KL, Newton HJ, Lührmann A, Roy CR. The *Coxiella burnetii* Dot/Icm system delivers a unique repertoire of type IV effectors into host cells and is required for intracellular replication. PLoS Pathog. 2011;7: e1002056 10.1371/journal.ppat.1002056 21637816 PMC3102713

[148] Várnai P, Lin X, Lee SB, Tuymetova G, Bondeva T, Spät A, et al Inositol lipid binding and membrane localization of isolated pleckstrin homology (PH) domains. Studies on the PH domains of phospholipase C δ1 and p130. J Biol Chem. 2002;277: 27412–27422. 10.1074/jbc.M109672200 12019260

[149] Várnai P, Rother KI, Balla T. Phosphatidylinositol 3-kinase-dependent membrane association of the Bruton’s tyrosine kinase pleckstrin homology domain visualized in single living cells. J Biol Chem. 1999;274: 10983–10989. 10.1074/jbc.274.16.10983 10196179

[150] Pattni K, Jepson M, Stenmark H, Banting G. A PtdIns(3)P-specific probe cycles on and off host cell membranes during *Salmonella* invasion of mammalian cells. Current Biology. 2001;11: 1636–1642. 10.1016/S0960-9822(01)00486-9 11676927

[151] Balla A, Tuymetova G, Tsiomenko A, Várnai P, Balla T. A plasma membrane pool of phosphatidylinositol 4-phosphate is generated by phosphatidylinositol 4-kinase type-III alpha: studies with the PH domains of the oxysterol binding protein and FAPP1. Mol Biol Cell. 2005;16: 1282–1295. 10.1091/mbc.E04-07-0578 15635101 PMC551492

[152] Knodler LA, Vallance BA, Hensel M, Jäckel D, Finlay BB, Steele-Mortimer O. *Salmonella* type III effectors PipB and PipB2 are targeted to detergent-resistant microdomains on internal host cell membranes. Mol Microbiol. 2003;49: 685–704. 12864852 10.1046/j.1365-2958.2003.03598.x

[153] Gietz RD, Schiestl RH, Willems AR, Woods RA. Studies on the transformation of intact yeast cells by the LiAc/SS-DNA/PEG procedure. Yeast. 1995;11: 355–360. 10.1002/yea.320110408 7785336

