# Supplementary figures and images for: SopF, a phosphoinositide binding effector, promotes the stability of the nascent Salmonella-containing vacuole
Source: PLoS Pathog. 2019 Jul 24;15(7):e1007959. doi: 10.1371/journal.ppat.1007959 (PMC6682159; doi:10.1371/journal.ppat.1007959)

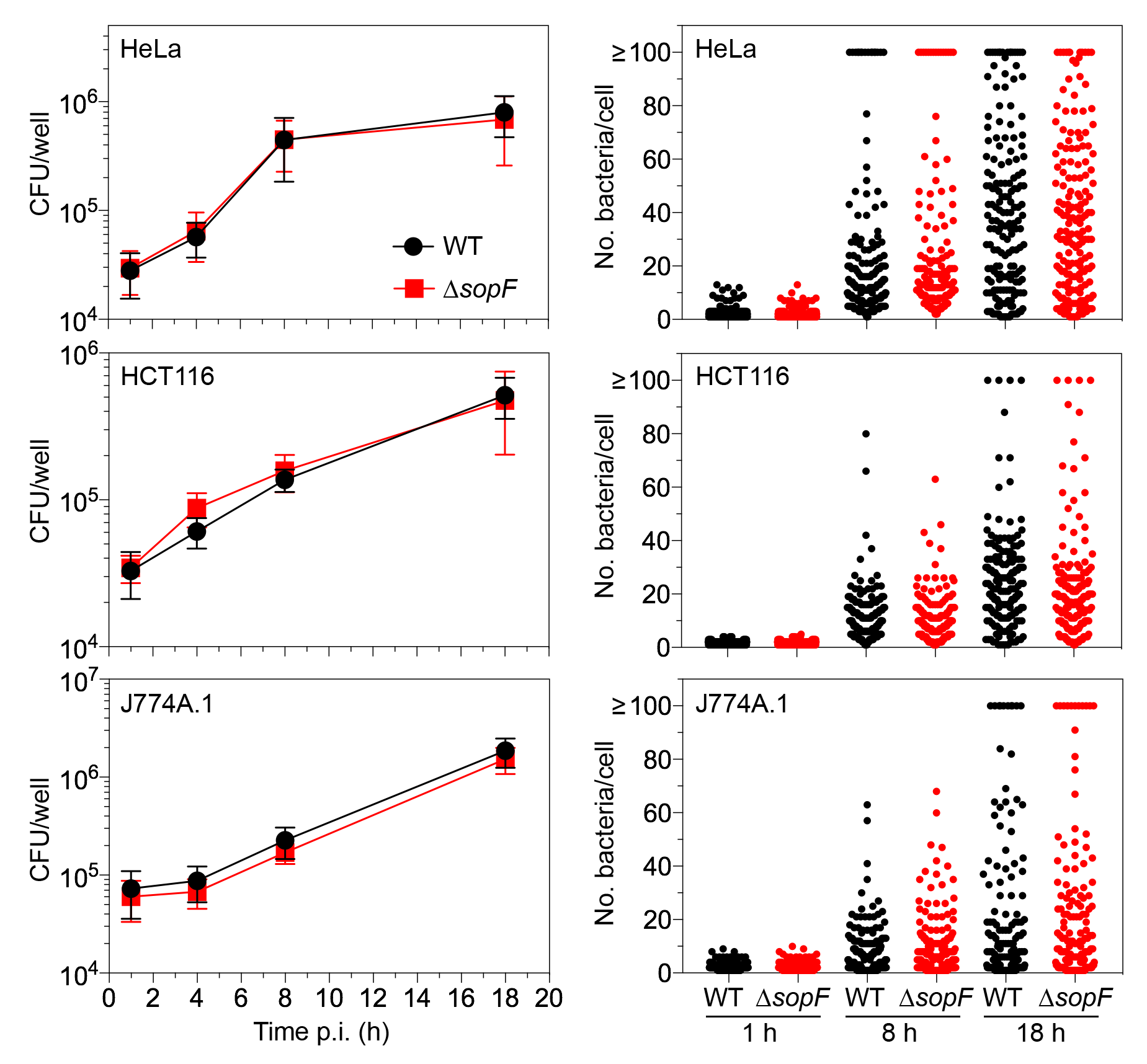

Supplement: S1 Fig — HeLa epithelial cells, HCT116 epithelial cells and J774A.1 macrophage-like cells were infected with S. Typhimurium wild type (WT) or ΔsopF bacteria. Bacterial replication was monitored by gentamicin protection assay (left panels) or fluorescence microscopy (right panels, mCherry expressing bacteria). Left panels, mean ± SD, n≥3 independent experiments; right panels, each dot represents one infected cell, data is combined from two independent experiments. (TIF) [file ppat.1007959.s001.tif]

A

EGFP-SopF

overlay

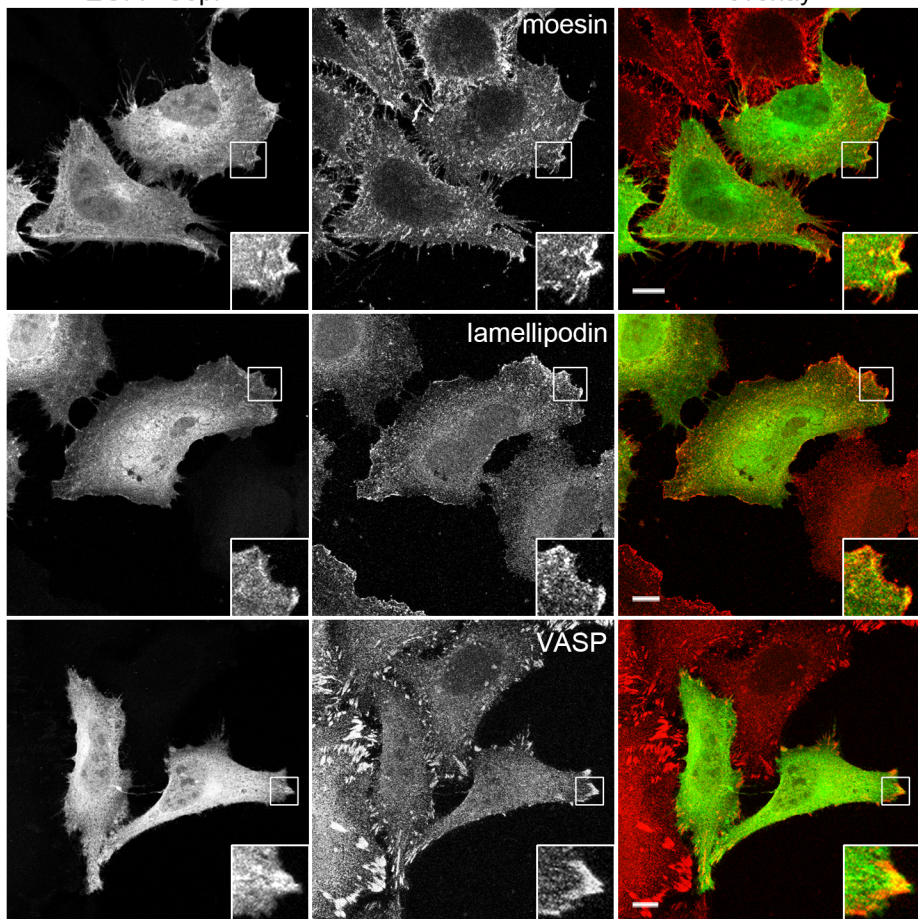

B

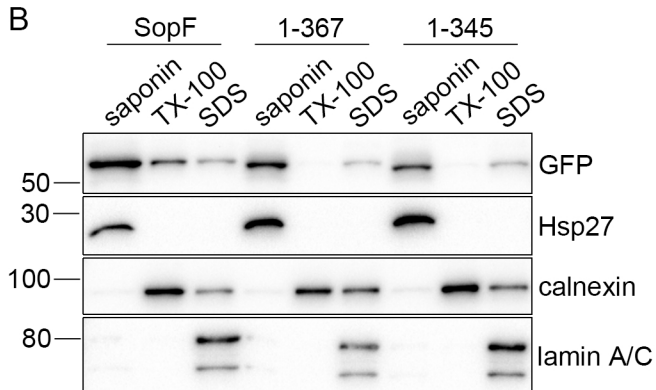

C

EGFP-SopF(1-367)

EGFP-SopF(1-345)

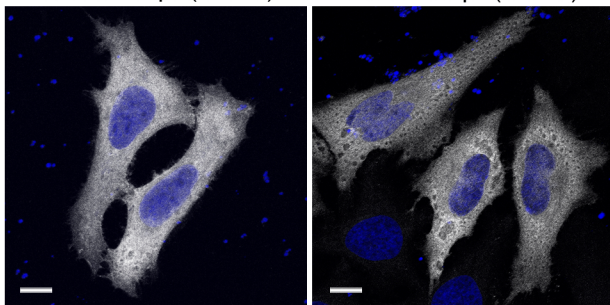

Supplement: S2 Fig — (A) EGFP-SopF partially colocalizes with actin-binding proteins found at cell adhesion sites. HeLa cells were transfected with EGFP-SopF for 18 h, then fixed and immunostained with anti-moesin, anti-lamellipodin and anti-vasodilator-stimulated phosphoprotein (VASP) antibodies. Representative confocal microscopy images show EGFP-SopF in green and moesin, lamellipodin or VASP in red. Scale bars are 10 μm. Insets show enlargements of boxed areas. (B) Membrane association depends on the carboxy-terminus of SopF. HeLa cells were transfected with plasmids encoding for EGFP-SopF, EGFP-SopF(1–367) or EGFP-SopF(1–345) for 18 h, then cells were collected and subject to sequential detergent fractionation. Equal volumes of saponin-soluble, TX-100-soluble and SDS-soluble fractions were separated by SDS-PAGE and subject to immunoblotting with antibodies against GFP, Hsp27 (cytosol), calnexin (membranes) and lamin A/C (nucleus). Molecular mass markers are indicated on the left. Results are representative of two independent experiments. (C) HeLa cells were transfected with plasmids encoding for EGFP-SopF(1–367) or EGFP-SopF(1–345) for 18 h. Cells were fixed and DNA was stained with Hoechst 33342. Representative confocal microscopy images show EGFP-SopF in greyscale and DNA in blue. Scale bars are 10 μm. (PDF) [file ppat.1007959.s002.pdf]

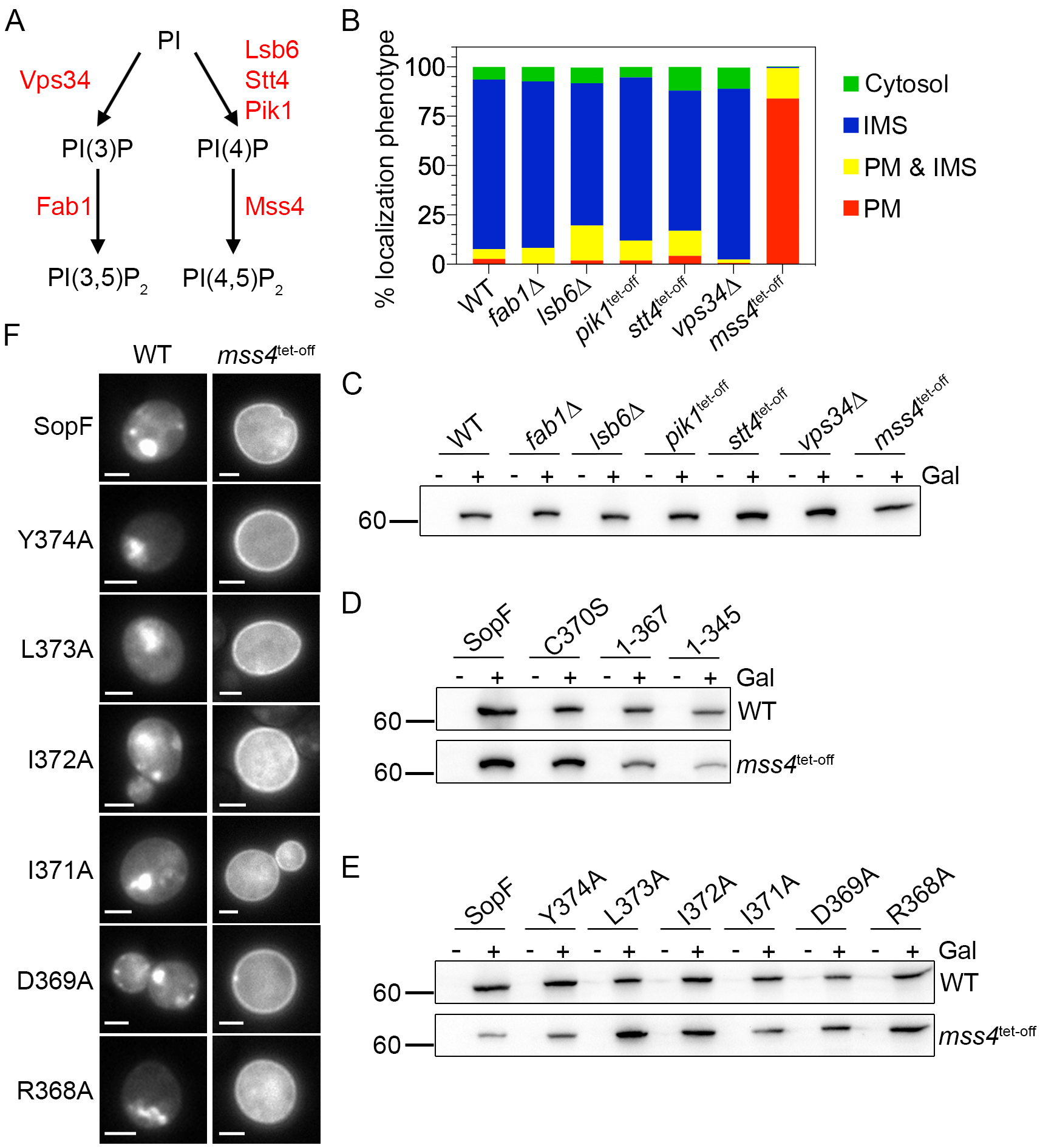

Supplement: S3 Fig — (A) Phosphoinositide synthesis in yeast is governed by six PI kinases (depicted in red). (B) The indicated yeast strains were transformed with yEGFP-SopF and its subcellular localization was visualized by widefield fluorescence microscopy and categorized as cytosol, internal membrane sites (IMS), plasma membrane (PM), or IMS and PM. Results are expressed as the mean percentage of total yeast transformants (n ≥ 200 cells from three independent transformations). (C, D, E) Immunoblot analysis of yEGFP-SopF production in wild type and PI kinase mutant yeast strains. Lysates were prepared from cells grown in the absence (-) or presence (+) of galactose (Gal). Proteins were subject to immunoblotting with anti-GFP antibodies. Molecular mass markers are indicated on the left. Results are representative of two independent experiments. (F) Wild type (WT) and mss4tet-off yeast strains were transformed with plasmids encoding for yEGFP-SopF and the indicated yEGFP-SopF point mutants. The subcellular localization of SopF in live cells was visualized by widefield fluorescence microscopy. Representative fluorescence images are shown. Scale bars are 2 μm. (TIF) [file ppat.1007959.s003.tif]

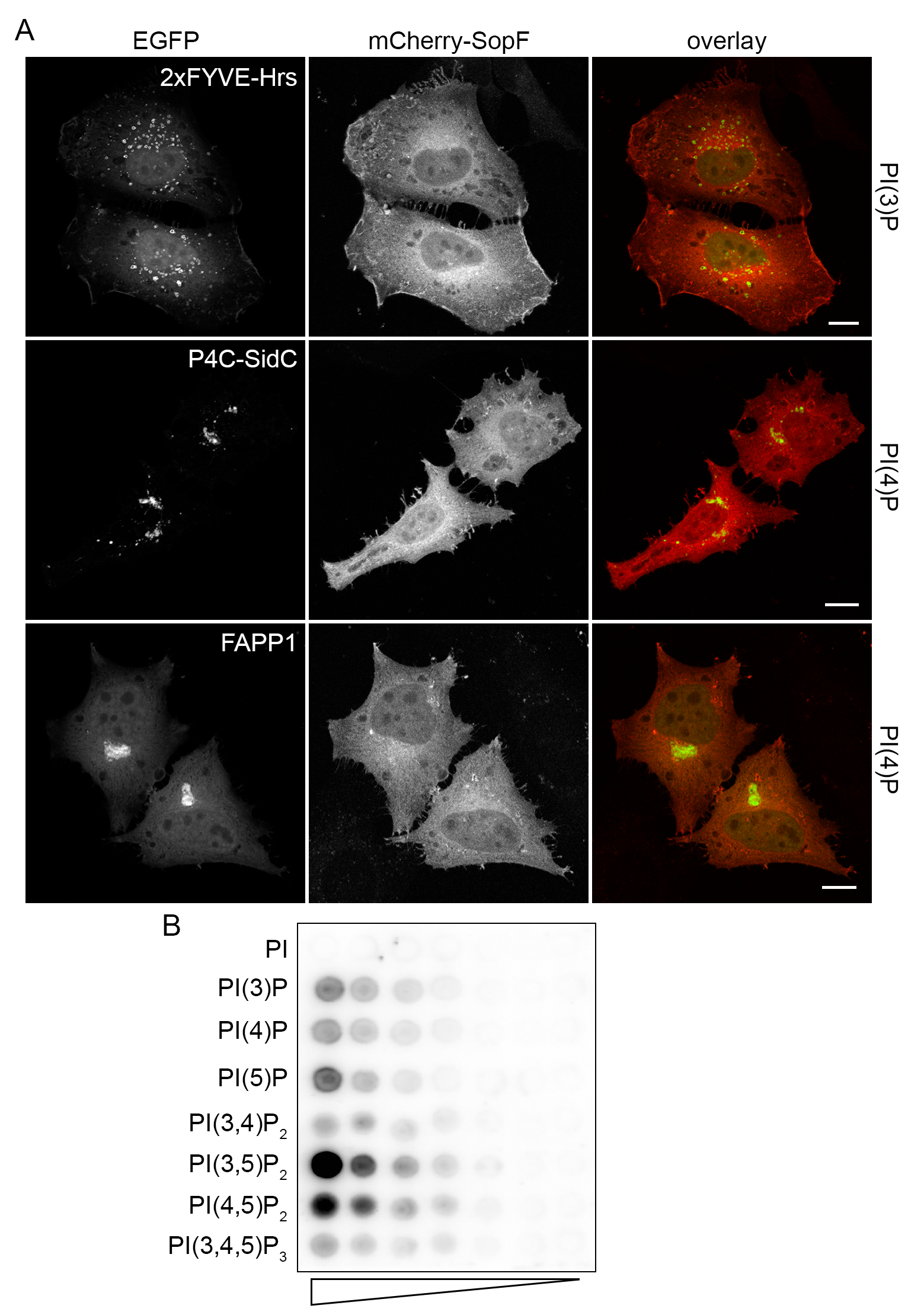

Supplement: S4 Fig — (A) SopF does not colocalize with phosphoinositide pools present on early endosomes or the Golgi. HeLa cells were co-transfected with mCherry-SopF and EGFP-phosphoinositide-binding domain chimeras for 16 h and fixed. Representative confocal microscopy images show phosphoinositide-binding probes in green and mCherry-SopF in red. PI(3)P on early endosomes is bound by 2xFYVE-Hrs and PI(4)P at the Golgi is bound by P4C-SidC and PH-FAPP1. Scale bars are 10 μm. (B) Protein-lipid overlay assay with SopF. Recombinant GST-SopF was purified by affinity chromatography and incubated with a PIP Array (Echelon Biosciences) at 1 μg/ml. Bound protein was detected using anti-GST antibodies followed by chemiluminescence detection. The following compounds are spotted on the nitrocellulose membrane in decreasing concentrations (100 pmol to 1.56 lipid per spot): phosphatidylinositol phosphate (PI); PI(3)P; PI(4)P; PI(5)P; PI(3,4)P2; PI(3,5)P2; PI(4,5)P2; PI(3,4,5)P3. (TIF) [file ppat.1007959.s004.tif]

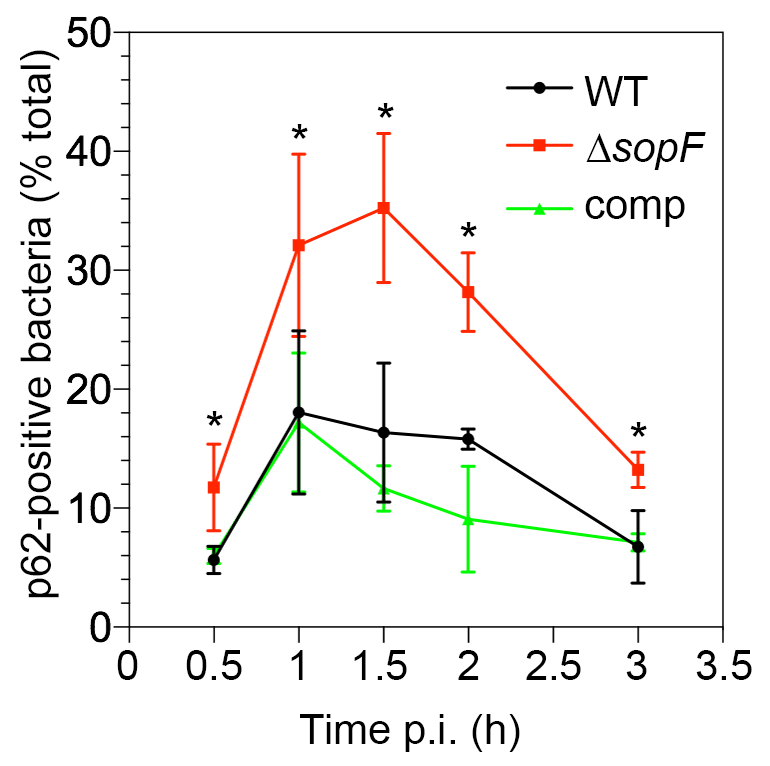

Supplement: S5 Fig — HeLa cells were infected with the following mCherry-expressing S. Typhimurium strains–wild type (WT), ΔsopF and ΔsopF pSopF-3xFLAG (comp). At the indicated times, cells were fixed and immunostained for the autophagy adaptor protein, p62/SQSTM1. The number of p62-positive bacteria was quantified by fluorescence microscopy. Data are the mean ± SD (n ≥ 3 experiments). Asterisks represent data significantly different to WT infection (one-way ANOVA with Dunnett’s post-hoc test). (TIF) [file ppat.1007959.s005.tif]

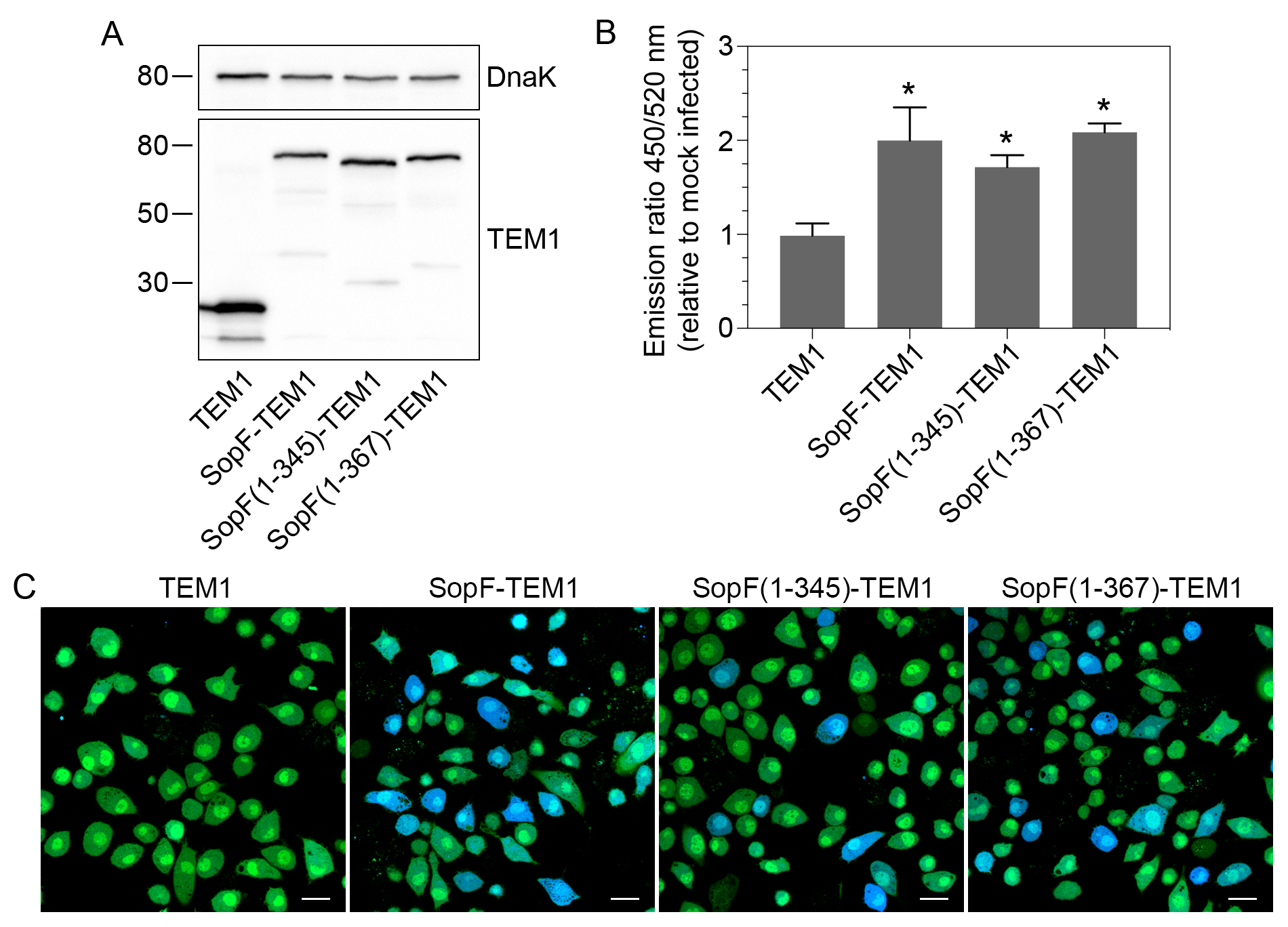

Supplement: S6 Fig — (A) Detection of TEM1 fusion proteins. Whole cell lysates from S. Typhimurium wild type harboring pCX340 (TEM1), pCX340-SopF (SopF-TEM1), pCX340-SopF(1–345) (SopF(1–345)-TEM1) or pCX340-SopF(1–367) (SopF(1–367)-TEM1) were subject to immunoblotting with antibodies against TEM1 β-lactamase and DnaK (loading control). Cultures were induced with 1 mM IPTG for 1 h prior to collection. Molecular mass markers are indicated on the left. (B) β-lactamase activity in infected cells. J774A.1 macrophage-like cells were infected with the S. Typhimurium strains indicated in (A), then cells were loaded with CCF2-AM substrate. At 2 h p.i., β-lactamase activity was detected by measuring cleavage of the CCF2-AM substrate on a fluorescence plate reader. Data is presented as the emission ratio between blue fluorescence (450 nm) and green fluorescence (520 nm). Ratios were normalized to that of mock infected cells in each experiment. Data are mean ± SD (3 independent experiments). Asterisks indicate data significantly different to TEM1 (one-way ANOVA with Dunnett’s post-hoc test). (C) Fluorescence microscopy detection of β-lactamase activity. J774A.1 cells were infected and loaded with CCF2-AM substrate as in (B). Blue fluorescence indicates CCF2-AM cleaved upon effector translocation, whereas uncleaved CCF2-AM emits a green fluorescence. Representative confocal images are shown. Scale bars are 20 μm. (TIF) [file ppat.1007959.s006.tif]
