# Supplementary material for: SopF, a phosphoinositide binding effector, promotes the stability of the nascent Salmonella-containing vacuole
Source: PLoS Pathog. 2019 Jul 24;15(7):e1007959. doi: 10.1371/journal.ppat.1007959 (PMC6682159; doi:10.1371/journal.ppat.1007959)
Supplement: S1 Table — (DOCX) [file ppat.1007959.s007.docx]

**S1 Table. Oligonucleotides used for cloning**

| **Name** | **Sequence (5’ to 3”)** | **Plasmid description** |
| --- | --- | --- |
| p413GalSL1177-Eco | GGAATTC**ATG**CTCAAACCTATCTGCCAT | p413Gal-yEGFP-SopF  p413Gal-yEGFP-SopF(1-367)  p413Gal-yEGFP-SopF(1-345)  p413Gal-yEGFP-SopF Y374A  p413Gal-yEGFP-SopF L373A  p413Gal-yEGFP-SopF I372A  p413Gal-yEGFP-SopF I371A  p413Gal-yEGFP-SopF C370S  p413Gal-yEGFP-SopF D369A  p413Gal-yEGFP-SopF R368A |
| p413GalSL1177-Sal | ACGCGTCGAC**TCA**ATATAATATTATGCAGTCTCT | p413Gal-yEGFP-SopF  mCherry-SopF |
| p413Gal-SopF345-R | ACGCGTCGAC**TCA**CAAAGACAAATAAACGTGTTTTAT | p413Gal-yEGFP-SopF(1-345)  EGFP-SopF(1-345) |
| p413Gal-SopF367-R | ACGCGTCGAC**TCA**ATTAAGCGCCTGGTTTATTTGTTT | p413Gal-yEGFP-SopF(1-367)  EGFP-SopF(1-367) |
| p413Gal-SopFR368A | ACGCGTCGAC**TCA**ATATAATATTATGCAGTC*TGC*ATTAAGCGCCTGGTT | p413Gal-yEGFP-SopF R368A |
| p413Gal-SopFD369A | ACGCGTCGAC**TCA**ATATAATATTATGCA*GGC*TCTATTAAGCGCCTG | p413Gal-yEGFP-SopF D369A |
| p413Gal-SopF_C370S_R_Sal | ACGCGTCGAC**TCA**ATATAATATTAT*GGA*GTCTCTATT | p413Gal-yEGFP-SopF C370S |
| p413Gal-SopFI371A | ACGCGTCGAC**TCA**ATATAATAT*TGC*GCAGTCTCTATTAAGCGC | p413Gal-yEGFP-SopF I371A |
| p413Gal-SopFI372A | ACGCGTCGAC**TCA**ATATAA*TGC*TATGCAAGTCTCTATTAAG | p413Gal-yEGFP-SopF I372A |
| p413Gal-SopFL373A | ACGCGTCGAC**TCA**ATA*TGC*TATTATGCAGTCTCTATT | p413Gal-yEGFP-SopF L373A |
| p413Gal-SopFY374A | ACGCGTCGAC**TCA***AGC*TAATATTATGCAGTCTCTATT | p413Gal-yEGFP-SopF Y374A |
| pEGFPC2-SopF-F | GGAATTC**ATG**CTCAAACCTATCTGCCAT | EGFP-SopF  EGFP-SopF(1-367)  EGFP-SopF(1-345) |
| pEGFPC2-SopF-R | CGGGATCC**TCA**ATATAATATTATGCAGTCTCT | EGFP-SopF |
| mCherryC1-SopF-F | CGGAATTCT**ATG**CTCAAACCTATCTGCCAT | mCherry-SopF |
| pcDNA4TOFLAG_SopF_F_BamHI | CGGGATCC**ATG**CTCAAACCTATCTGCCAT | FLAG-SopF  pGEX-6P-1-SopF |
| pcDNA4TOFLAG_SopF_R_EcoRI | AAGAATTC**TCA**ATATAATATTATGCAGTCTCT | FLAG-SopF  pGEX-6P-1-SopF |
| pcDNA4TOFLAG_SopF367_R_EcoRI | CGGAATTC**TCA**ATTAAGCGCCTGGTTTATTTGTTTT | FLAG-SopF(1-367) |
| pcDNA4TOFLAG_SopF345_R_EcoRI | CGGAATTC**TCA**CAAAGACAAATAAACGTGTTTTATAT | FLAG-SopF(1-345) |
| SL1177-3xFLAG-for | ATAAACCAGGCGCTTAATAGAGACTGCATAATATTATATGACTACAAAGACCATGACGG | *sopF*::3xFLAG |
| SL1177-3xFLAG-rev | AGCCAGCAGGCGCTTCTGCTGGTCCATATTCCTCTACATATGAATATCCTCCTTAG | *sopF*::3xFLAG |
| Sma-SL1177CyaA-F | TCCCCCGGGACTTAACGTCCCCGTTTTTAT | pSopF-CyaA  pSopF-3xFLAG  pSopF-2xHA  pSopF  pSopF(1-345)  pSopF(1-367) |
| SL1177CyaA-OLF | GCAGATTCACCTTATGCCCCATATCAGCAATCGCATCAGGCTGGTTAC | pSopF-CyaA |
| SL1177CyaA-OLR | GTAACCAGCCTGATGCGATTGCTGATATGGGGCATAAGGTGAATCTGC | pSopF-CyaA |
| Xho-SL1177CyaA-R | CCGCTCGAG**CTA**GCGTTCCACTGCGCCCAG | pSopF-CyaA |
| Xho-SL1177FLAG-R | CCGCTCGAG**CTA**TTTATCGTCGTCATCTTTGTA | pSopF-3xFLAG |
| Xho-SL11772HA-R | CCGCTCGAG**TCA**CGCATAATCCGGCACATCATACGGATACGCATAATCCGGCACATCATACGGATATAATATTATGCAGTCTCTATTAAG | pSopF-2xHA |
| Xho-SL1177-R | CCGCTCGAG**TCA**ATATAATATTATGCAGTC | pSopF |
| Xho-SL1177-345R | CCGCTCGAG**TCA**CAAAGACAAATAAACGTGTTT | pSopF(1-345) |
| Xho-SL1177-367R | CCGCTCGAG**TCA**ATTAAGCGCCTGGTTTATTTGTTT | pSopF(1-367) |
| Xba-dSL1177-F1 | GCTCTAGAGGCTCAGGTTGGCGGTATCGG | pRE112-∆*sopF* |
| Sac-dSL1177-R2 | CCGAGCTCCATCAATTGGTGTGATATCA | pRE112-∆*sopF* |
| dSL1177-OL-F2 | TTCAGGAGACAT**ATG**CTCTAT**TGA**CAAGCTTATTATCAGTCT | pRE112-∆*sopF* |
| dSL1177-OL-R1 | ATAAGCTTG**TCA**ATAGAG**CAT**ATGTCTCCTGAATTTTTATGACTA | pRE112-∆*sopF* |
| pCX340-SopF-F | AAGGTACC**ATG**CTCAAACCTATCTGCCAT | pSopF-TEM1  pSopF(1-367)-TEM1  pSopF(1-345)-TEM1 |
| pCX340-SopF-R | AAGAATTCTCATATAATATTATGCAGTCTCT | pSopF-TEM1 |
| pCX340-367-R | AAGAATTCTCATTAAGCGCCTGGTTTATTTGTTT | pSopF(1-367)-TEM1 |
| pCX340-345-R | AAGAATTCTCCAAAGACAAATAAACGTGTTTTAT | pSopF(1-345)-TEM1 |

Engineered restriction sites are underlined. Start and stop codons are in **bold**. Amino acid point mutations are *italicized*.
